# Highly efficient single-stranded DNA ligation technique improves low-input whole-genome bisulfite sequencing by post-bisulfite adaptor tagging

**DOI:** 10.1093/nar/gkz435

**Published:** 2019-05-22

**Authors:** Fumihito Miura, Yukiko Shibata, Miki Miura, Yuhei Sangatsuda, Osamu Hisano, Hiromitsu Araki, Takashi Ito

**Affiliations:** 1 Department of Biochemistry, Kyushu University Graduate School of Medical Sciences, 3-1-1 Maidashi, Higashi-ku, Fukuoka 812-8582, Japan; 2 Precursory Research for Embryonic Science and Technology (PRESTO), Japan Science and Technology Agency (JST), 3-1-1 Maidashi, Higashi-ku, Fukuoka 812-8582, Japan

## Abstract

Whole-genome bisulfite sequencing (WGBS) is the current gold standard of methylome analysis. Post-bisulfite adaptor tagging (PBAT) is an increasingly popular WGBS protocol because of high sensitivity and low bias. PBAT originally relied on two rounds of random priming for adaptor-tagging of single-stranded DNA (ssDNA) to attain high efficiency but at a cost of library insert length. To overcome this limitation, we developed terminal deoxyribonucleotidyl transferase (TdT)-assisted adenylate connector-mediated ssDNA (TACS) ligation as an alternative to random priming. In this method, TdT attaches adenylates to the 3′-end of input ssDNA, which are then utilized by RNA ligase as an efficient connector to the ssDNA adaptor. A protocol that uses TACS ligation instead of the second random priming step substantially increased the lengths of PBAT library fragments. Moreover, we devised a dual-library strategy that splits the input DNA to prepare two libraries with reciprocal adaptor polarity, combining them prior to sequencing. This strategy ensured an ideal base–color balance to eliminate the need for DNA spike-in for color compensation, further improving the throughput and quality of WGBS. Adopting the above strategies to the HiSeq X Ten and NovaSeq 6000 platforms, we established a cost-effective, high-quality WGBS, which should accelerate various methylome analyses.

## INTRODUCTION

The methylome is a genome-wide distribution of 5-methylcytosine, and its patterns are often specific for cells and tissues but dynamically modulated in response to various stimuli and environmental changes. Whole-genome bisulfite sequencing (WGBS) has made it possible to draw an almost complete picture of the methylome at single-nucleotide resolution (1,2), which has led to various novel findings of biomedical importance. However, the initial protocols for WGBS library preparation required microgram quantities of input DNA (1,2) and were hence not readily applicable to samples whose quantities were limited. Accordingly, various efforts have been made to improve the efficacy of library preparation to expand the WGBS targets. For example, Tn5mC-seq used Tn5 transposase-mediated tagmentation, an extremely efficient adaptor tagging method for dsDNA, to successfully reduce the amount of input DNA to tens of nanograms (3). However, even with the ultimate efficiency of adaptor tagging, Tn5mC-seq still required global amplification of the library with more than ten cycles of polymerase chain reaction (PCR) (3).

We independently investigated the cause of low yields in WGBS library preparation. Since it had been well documented that the mass yield of bisulfite treatment is fairly low (4–7), we first thought that the poor yield of library was attributed to the low yields of bisulfite treatment. However, to our surprise, the mass yields of DNA prepared with commercially available bisulfite treatment kits were not so low, ranging from 30 to 70% (8). Therefore, the major cause of the low yields in WGBS library preparation is not the mass yields of bisulfite treatment. On the contrary, we found that the order of operations (i.e. adaptor-tagging followed by bisulfite treatment) shared by all WGBS protocols, including MethylC-seq (2) and Tn5mC-seq (3), was the primary cause of low efficiency of library preparation. This is because bisulfite-induced DNA degradation leads to a drastic reduction of intact library molecules (i.e. genomic DNA sandwiched by two adaptors, Supplementary Figure S1) (8). To avoid this adverse effect of bisulfite treatment, we have proposed an alternative strategy, termed post-bisulfite adaptor tagging (PBAT), in which adaptor tagging is performed after bisulfite treatment.

Because bisulfite-treated DNA is single-stranded, the implementation of PBAT requires an efficient method for the attachment of adaptor sequences to single-stranded DNA (ssDNA). Accordingly, we deployed two rounds of random priming in the original implementation of the PBAT protocol (8), referred to hereafter as random priming-mediated PBAT (rPBAT). Optimized random priming was so efficient that rPBAT enabled WGBS library preparation from as little as 125 pg of genomic DNA (8). Indeed, rPBAT enabled WGBS from only a few thousands of mouse eggs and primordial germ cells, revealing their methylomes for the first time, notably, without any global PCR amplification (9,10). The method was also successfully applied to a limited amount of DNA fragments enriched by solution hybridization capture for highly sensitive targeted methylome sequencing (11). With iterated random priming followed by PCR amplification, rPBAT has even been used in single-cell methylome sequencing, albeit with compromised genomic coverage (12). Moreover, recent systematic analysis of WGBS protocols revealed that amplification-free rPBAT provides the most accurate and the least biased WGBS data to date (13).

The efficiency of library preparation by rPBAT is higher than those of conventional procedures, including MethylC-Seq (2) and Tn5mC-Seq (3). Nevertheless, rPBAT converts only ∼10% of the input DNA to library fragments, thus leaving room for further improvement. It has been shown that iterated random priming of bisulfite-converted DNA enhances the efficacy of library preparation for single-cell methylome analysis (12), but at the cost of increased fraction of unmappable reads (∼80%). Indeed, rPBAT libraries, especially those prepared from smaller amounts of DNA (<250 pg), contain increasing amounts of unmappable reads (8). In addition, we have shown that the rPBAT library contains a sizable fraction of chimeric reads (8). All these drawbacks of rPBAT are likely attributable to the random priming reaction, as follows. First, random priming usually starts not at the 3′-terminus but at a site within the target ssDNA fragment, thereby leaving a sequence margin that is not included in the newly-synthesized strand to be sequenced. Thus, each library fragment is inevitably shorter than its target ssDNA, especially when two rounds of random priming are employed. Second, the efficiency of random priming depends on the stability of the random primer–genomic DNA duplex. Consequently, the random priming reaction often leads to a bias toward the GC-rich regions, with less efficient genomic coverage of the AT-rich regions. Third, random priming occurs not only between a primer and genomic DNA but also between two primers, as well as between two genomic DNA fragments, thus generating unmappable and chimeric reads, respectively. Therefore, it is critical to eliminate either or both of the random priming steps to further improve PBAT by extending the length of library fragments, mitigating GC-biased coverage, and reducing unmappable and chimeric reads.

Two approaches would be plausible to substitute a random priming reaction for adaptor-tagging of ssDNA. One is ssDNA ligation catalyzed by RNA ligase, which joins a 5′-phosphorylated adaptor to a 3′-end of ssDNA (14,15). Although RNA ligase very efficiently ligates the 5′-end of donor oligodeoxyribonucleotide (ODN) to the 3′-end of acceptor RNA, its ability to ligate donor ODN to the 3′-end of acceptor DNA is limited (16). It is thus not practical to use RNA ligase in adaptor-tagging of ssDNA. The other approach is terminal deoxyribonucleotidyl transferase (TdT)-mediated attachment of a homopolymeric tail to the 3′-end of ssDNA (17,18). The homopolymeric tail serves as a priming site for an adaptor-primer that contains a sequence complementary to the homopolymer to synthesize a complementary strand of ssDNA. TdT-mediated tailing is highly efficient, but a lengthy tail can constitute an obstacle for downstream processing. Hence, controlling the length of the homopolymer is critical. Interestingly, the length of a homopolymeric tail attached by TdT differs depending on whether deoxyribonucleotide triphosphates (dNTPs) or ribonucleotide triphosphates (NTPs) are used as its substrates. When dNTPs are used, TdT-mediated tailing is so processive that it is difficult to control the length of the tail. By contrast, when NTPs are used, the reaction notably terminates in a self-limiting manner or after addition of 2–4 residues (19).

The unique features of RNA ligase and TdT described above led us to devise a novel combinatorial application of these two enzymes for ssDNA ligation. We reasoned that if TdT attached a few ribonucleotides (e.g. AMP) to the 3′-end of ssDNA, then RNA ligase would use them as an efficient ‘connector’ to 5′-phosphorylated ODN. Based on this idea, we developed a highly efficient ssDNA ligation method, termed TdT-assisted adenylate connector-mediated ssDNA (TACS) ligation. Further, we used TACS ligation instead of the second random priming step of PBAT to successfully increase the length of PBAT library fragments and, hence, the throughput. We also developed a unique dual-library strategy to overcome the color imbalance problem inherent to WGBS and hence eliminate the need for DNA spike-in for color compensation, thereby further increasing the throughput of WGBS. Consequently, by adopting the dual-library TACS ligation-mediated PBAT (tPBAT) for the HiSeq X Ten and NovaSeq 6000 platforms, we have established a cost-effective, high-quality WGBS protocol.

## MATERIALS AND METHODS

### RNA ligases

T4 RNA ligase was purchased from Takara Bio Inc. (Shiga, Japan). Mth RNA ligase was a component of the 5′ DNA adenylation kit from New England Biolabs (Ipswich, MA, USA). Thermostable 5′AppDNA/RNA Ligase and T4 RNA ligase 2 were also from New England Biolabs. TS2126 RNA ligase and RM378 RNA ligase were prepared in-house (for details, see Supplementary Methods).

### ODNs

ODNs used in the current study were synthesized by Eurofins Genomics (Tokyo, Japan) with oligonucleotide purification cartridge-grade purification. Their sequences with modifications and the combinations to be used for tPBAT protocols are summarized in Supplementary Tables S1–S3.

### TACS ligation

A solution was prepared containing 10 μl of 2.5 × TACS reaction buffer [125 mM HEPES–KOH, pH 7.5, 12.5 mM MgCl_2_, 1.25% (v/v) Triton-X100, and 50% (w/v) polyethylene glycol (PEG) 6000 (Nacalai Tesque, Kyoto, Japan)], 1 μl of 10 mM adenosine triphosphate (ATP) and appropriate amounts of acceptor and dual-phosphorylated adaptor (see Supplementary Table S3) and the total volume was adjusted to 23 μl. Using a thermal cycler, the reaction was denatured at 95°C for 3 min then cooled to 4°C. Next, the reaction was supplemented with 1 μl each of 15 U/μl TdT (Takara Bio Inc.) and 2 mg/ml TS2126 RNA ligase. The reaction mixture was sequentially incubated at 37°C for 30 min, 65°C for 1 h and 95°C for 5 min.

### Denaturing polyacrylamide gel electrophoresis

DNA fragments were analyzed on denaturing polyacrylamide gel electrophoresis using 6% Novex TBE Urea gel (Thermo Fisher Scientific, Waltham, MA). After electrophoresis, the gel was stained with SYBR Gold gel stain (Thermo Fisher Scientific) and photographed with ChemiDoc Touch (Bio Rad Laboratories, Hercules, CA). Quantitation was performed using Image Lab 5.2 (Bio Rad Laboratories).

### tPBAT protocol

#### Bisulfite treatment

Bisulfite treatment of genomic DNA was performed using EZ DNA Methylation Gold kit (Zymo Research, Irvine, CA, USA). If necessary, bisulfite conversion rate was monitored by spiked-in unmethylated lambda DNA (Promega, Fitchburg, WI, USA), which constituted 1% of the sample DNA. The bisulfite treatment yields were monitored using Qubit fluorometer and Qubit ssDNA assay kit (Thermo Fisher Scientific).

#### First strand synthesis

The following were added to the bisulfite-treated DNA prepared as above: 5 μl of 10 × NEBuffer 2 (New England Biolabs), 5 μl of 2.5 mM dNTPs (Takara Bio Inc) and 100 pmol of random primer (see Supplementary Table S3); the reaction volume was adjusted to 50 μl with water. The mixture was incubated at 95°C for 5 min, and then cooled and kept at 4°C for 5 min. To the reaction, 1 μl of 50 U/μl Klenow fragment (3′–5′ exo minus) (New England Biolabs) was added and the mixture was incubated at 4°C for 15 min. The reaction temperature was gradually raised to 37°C at a rate of 1°C/min and then maintained at 37°C for 30 min. After heat inactivation of the enzyme at 70°C for 10 min, 50 μl of AMPure XP (Beckman Coulter, Brea, CA, USA) was added, and the reaction mixture was incubated at room temperature for 5 min. The supernatant was removed on a magnetic stand, the beads were rinsed with 75% ethanol and purified DNA was eluted with 13 μl of 10 mM Tris–acetate, pH 8.0. The yield of first-strand synthesis was monitored using Qubit and Qubit dsDNA HS assay kit (Thermo Fisher Scientific), following the manufacturer's instructions.

#### TACS ligation

The following were added to 12 μl of purified first-strand synthesis product: 10 μl of 2.5 × TACS reaction buffer, 1 μl of 10 mM ATP and 1 μl of 5 μM dual-phosphorylated adaptor (see Supplementary Table S3). The reaction was performed as described above.

#### Synthesis of complementary strand to the adaptor-tagged DNA

The following were added to 25 μl of TACS ligation product: 5 μl of 10 × ExTaq buffer (Takara Bio Inc.), 5 μl of 2.5 mM dNTPs (Takara Bio Inc.), 10 pmol of second strand primer (see Supplementary Table S1) and 2.5 U of hot-start Gene Taq (Nippon Gene, Toyama, Japan); the total volume was adjusted to 50 μl with water. The reaction mixture was sequentially incubated at 95°C for 1 min, 45°C for 3 min and 72°C for 3 min. Then, solid phase reversible immobilization (SPRI)-based purification (20) was performed as follows. To the reaction, 2 μl of Sera-Mag carboxylate (GE Healthcare, Pittsburgh, PA, USA), 2 μl of 1 M MgCl_2_ and 50 μl of 100% ethanol were added, and the mixture was incubated at room temperature for 5 min. After removal of the supernatant on a magnetic stand, the beads were rinsed with 75% ethanol. Purified DNA was eluted with 20 μl of 10 mM Tris–acetate, pH 8.0. If necessary, the yields of dsDNA were monitored using Qubit and Qubit dsDNA HS assay kit (Thermo Fisher Scientific), following the manufacturer's instructions.

#### Final primer extension

The following were added to 20 μl of double-stranded library: 5 μl of 10 × ExTaq buffer (Takara Bio Inc), 5 μl of 2.5 mM dNTPs, 10 pmol of the final primer (see Supplementary Table S1) and 2.5 U of hot-start Gene Taq; the total volume was adjusted to 50 μl with water. The reaction mixture was sequentially incubated at 95°C for 1 min, 45°C for 3 min and 72°C for 3 min. Next, 50 μl of AMPure XP (Beckman Coulter, Brea, CA, USA) was added, and the mixture was incubated at room temperature for 5 min. The supernatant was removed on a magnetic stand, and the beads were washed three times with 200 μl of the cut-off solution [10 mM Tris–HCl, pH 8.0, 1 M NaCl, and 19% (v/v) PEG400 (Nacalai Tesque)] and at last rinsed with 75% ethanol. Purified DNA was eluted with 20 μl of 10 mM Tris–acetate, pH 8.0. The yields of eluted DNA were determined by quantitative PCR (qPCR) using a library quantification kit (Takara Bio Inc.) according to the manufacturer's instructions. The size distribution of amplified PCR products was analyzed on denaturing polyacrylamide gel electrophoresis using 6% Novex TBE Urea gel as described above.

### Next generation sequencing

Small-scale sequencing was performed using the Illumina MiSeq system with the reagent kit version 3 (150 cycles) and the reagent nano kit version 2 (300 cycles) (San Diego, CA, USA), with some modifications for denaturation and neutralization of the library, as described previously (8). The amount of input library varied depending on the purpose of the experiments. Large-scale sequencing using the HiSeq X Ten and NovaSeq 6000 were performed by Macrogen Japan Corp. (Kyoto, Japan) as commercial services. Sequenced reads were delivered to us after demultiplexing of indexed libraries.

### Bioinformatics analysis

The sequenced reads were analyzed using a cluster system composed of 16 calculation nodes and network file system-based shared file storage. Univa Grid Engine was used for job scheduling. In-house programs were used for mapping and summarizing the sequenced reads. First, reads in fastq format were mapped to the reference genome composed of both human genome assembly GRCh37 (hg19) and enterobacteria phage lambda (GenBank: J02459) using BMap (http://itolab.med.kyushu-u.ac.jp/DT/BMap/). Alignments exported from BMap were summarized in a binary file in a custom format called the ‘graph file’ using MPTC (http://itolab.med.kyushu-u.ac.jp/DT/MPTC/). Mapping statistics were calculated using MapSum (http://itolab.med.kyushu-u.ac.jp/DT/MapSum/) and the basic properties of the methylome data were calculated using CalcCoverage (http://itolab.med.kyushu-u.ac.jp/DT/CalcCoverage/). The source code of these programs can be downloaded from GitHub (https://github.com/FumihitoMiura/Project-2).

## RESULTS

### TACS ligation

**Figure 1.**
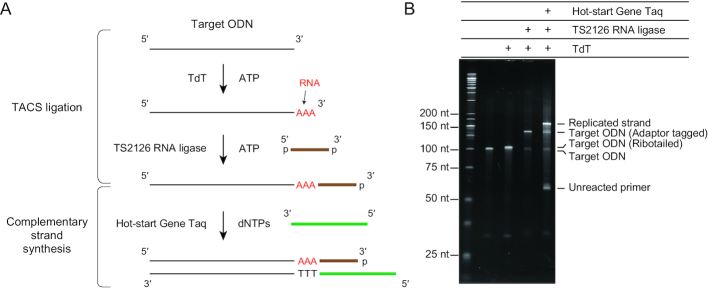
TACS ligation followed by complementary DNA synthesis. (**A**) The scheme for adaptor tagging using TACS ligation and synthesis of a strand complementary to the adaptor-tagged template. (**B**) Efficiencies of the two reactions, TACS ligation and complementary DNA synthesis, using model ODNs under optimized reaction conditions. For each reaction step, an equivalent amount of reaction was sampled. After denaturing polyacrylamide gel electrophoresis and SYBR Gold staining, a fluorescent image was obtained and signal intensities were calculated. A representative image of three independent experiments is presented.

Although T4 RNA ligase shows no preference for DNA or RNA as a donor, it much prefers RNA to DNA as an acceptor (16). These observations compelled us to ask whether the 3′-end of ssDNA being tailed with a few ribonucleotide residues forming the ‘ribotail’ could serve as an efficient connector to 5′-phosphorylated ssDNA in RNA ligase-mediated ligation (Figure 1A). We focused on TdT because it can add a few ribonucleotides to the 3′-end of ssDNA, notably, in a self-limiting manner (19). As expected, pre-treatment of acceptor ssDNA with TdT and ATP resulted in a remarkable enhancement of its ligation of a 5′-phosphorylated or 5′-adenylated donor ODN by commercially available RNA ligases (Supplementary Figures S2 and 3). These findings indicated the potential of a combinatorial use of TdT and RNA ligase as a method for adaptor-tagging of ssDNA.

We then optimized various reaction conditions for the combinatorial use of the two enzymes. As demonstrated for T4 RNA ligase (21), PEG enhanced the ligation efficiency (Supplementary Figure S3), especially when high-molecular weight PEG was used (Supplementary Figure S4). We evaluated both commercially available (T4 RNA ligase, T4 RNA ligase 2, Mth RNA ligase and 5′AppDNA/RNA ligase) and in-house prepared RNA ligases (RM378 RNA ligase (22) and TS2126 RNA ligase (23)) for use with TACS ligation (Supplementary Figures S3 and 5). We decided to use TS2126 RNA ligase throughout this study because it exhibited the highest ligation efficiency among the enzymes tested (Supplementary Figure S5). Of note, CircLigase II was able to replace TS2126 RNA ligase without reduction in reaction efficiency. Although TdT showed biased ribotailing efficiency dependent on the nucleobase at the 3′-termini of substrate ssDNA in some conditions (not shown), optimization of reaction conditions made the efficacy almost complete and, thus, virtually overcame the bias. Under the optimized condition, TACS ligation appeared to be barely affected by the size of acceptor DNA (Supplementary Figure S6) and the nucleobase at the 3′-end of the acceptor (Supplementary Figure S7). Although four nucleobases (i.e. A, C, G and U) served as largely comparable substrates for TdT, A outperformed the other three as the connector in RNA ligase-mediated ligation (Supplementary Figure S8). We also observed that PEG does not inhibit TdT activity (Supplementary Figure S9). Consequently, two reactions (i.e. ribotailing and ligation) could be sequentially conducted in a single reaction mixture if ATP was provided as the ribonucleotide substrate. We termed this method TACS ligation (Figure 1A). Notably, the efficiency of optimized TACS ligation was high in a model system (72.9 ± 2.8%, Figure 1B), thus demonstrating its excellent potential as a novel ssDNA ligation method.

### Synthesis of DNA complementary to adenylate-connected ssDNA

Once the 3′-end of target ssDNA is ligated to an adaptor via adenylates, DNA complementary to the target ssDNA can be readily synthesized by DNA polymerase using a primer complementary to the adaptor sequence. However, the products of TACS ligation contain 2–4 adenylates at the junction between the target and adaptor ssDNAs. Consequently, DNA polymerase used for complementary DNA synthesis has to be able to reverse-transcribe the connector adenylates. Although viral reverse transcriptases (RTases) can be used for this purpose, most of these enzymes are inactivated at the high temperatures that ensure highly stringent primer annealing and extension. We thus evaluated the use of thermostable DNA polymerases with RTase activity. We first tested KlenTaq M1, a Taq DNA polymerase variant with a weak RTase activity (24). Using model templates containing 2, 3 or 4 adenylates upstream of the primer-annealing site (Supplementary Figure S10A), we performed primer extension assays to determine whether the RTase activity of KlenTaq M1 can read through the short RNA stretch (Supplementary Figure S10B). In addition, ExTaq was used as a negative control or an enzyme with no-RTase activity. Unexpectedly, however, ExTaq showed activity comparable to that of KlenTaq M1 (Supplementary Figure S10B). This finding prompted us to investigate whether other DNA polymerases exhibit RTase activity enough to read through the short stretch of adenylates. While commercially available DNA polymerases, such as Phusion, KOD and PrimeStar, failed to extend the complementary strand of the short RNA stretch (Supplementary Figure S10C), Taq DNA polymerase and all its derivatives tested retained enough activity for reverse transcription of the short adenylate stretch (Supplementary Figure S10D). From these enzymes, we chose hot-start Gene Taq because it lacks the 5′-to-3′ exonuclease activity, while exhibiting a superior ability to use the short RNA stretch as its template. Because high PEG concentration optimal for TACS ligation inhibited Taq DNA polymerase and its derivatives (not shown), the ligation product had to be diluted before complementary DNA synthesis. After optimization, the efficiency of synthesis of DNA complementary to an adenylate-connected DNA reached almost 70% (69.5 ± 8.6%, Figure 1B), and the overall efficiency of the two reactions (i.e. TACS ligation and complementary strand synthesis) was calculated to be 50.2%.

### Elongation of WGBS library fragments by TACS ligation-mediated PBAT

**Figure 2.**
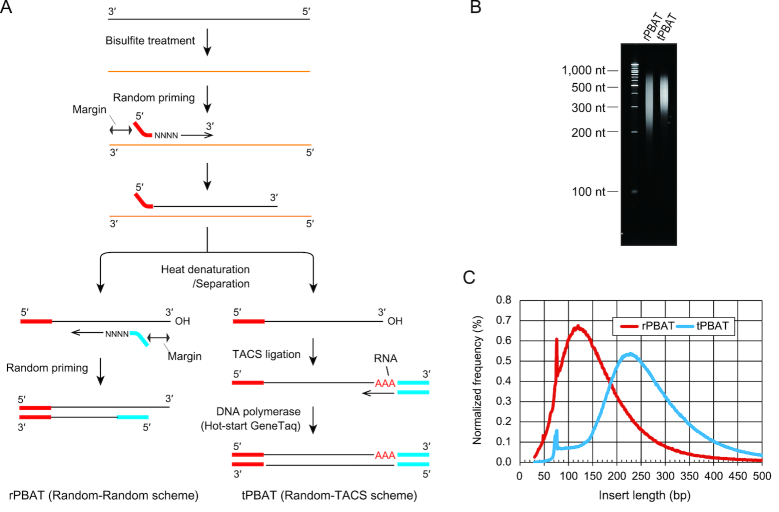
Comparison of library fragment lengths between rPBAT and tPBAT. (**A**) After the first round of random priming, a second round of random priming is performed in rPBAT (left), while TACS ligation-mediated adaptor ligation followed by replication of the adaptor-tagged template is performed in tPBAT (right). (**B**) Comparison of the size distribution of libraries prepared from genomic DNA extracted from the IMR90 cell line using rPBAT and tPBAT by denaturing polyacrylamide gel electrophoresis after PCR amplification of the libraries. Because libraries prepared using the original rPBAT protocol (8) cannot be used for paired-end sequencing, another rPBAT protocol that is compatible with paired-end sequencing (11) was used in the experiments. (**C**) Distribution of end-to-end distance of correctly mapped paired-end reads on the reference genome.

The original rPBAT protocol relies on two rounds of random priming for efficient adaptor-tagging of bisulfite-converted ssDNA. Because random primers rarely hybridize to the 3′-end of target ssDNA, it is not possible to synthesize a complementary DNA that covers the full-length of bisulfite-converted DNA fragment. Furthermore, two rounds of random priming inevitably render each library fragment much shorter than its cognate bisulfite-converted genomic DNA fragment (Figure 2A, left). To increase the length of library fragments, we employed TACS-ligation followed by complementary strand synthesis to substitute for the second random priming step of rPBAT (Figure 2A, right). Because the target DNA of TACS ligation should be single-stranded, the product of the first random priming step was heat-denatured before adaptor-tagging by TACS ligation. As expected, TACS ligation-mediated PBAT (tPBAT) generated longer library fragments than rPBAT, as indicated by the analysis of PCR-amplified library fragments (Figure 2B). This result was consolidated by paired-end sequencing of these libraries and subsequent calculation of the genomic distance between correctly mapped paired-end reads (Figure 2C). Hence, TACS ligation successfully increased the length of PBAT library fragments.

### Improvement of net WGBS throughput by a dual-library strategy

Because bisulfite treatment converts most cytosines (i.e. unmethylated cytosines) to uracils (4), the nucleotide composition of WGBS library is extremely biased (Supplementary Table S4). Accordingly, the total signal intensity corresponding to cytosines in conventional WGBS libraries or guanines in PBAT libraries declines to only a few percent of genomic DNA libraries without bisulfite treatment. Such unbalanced signal intensity often causes low read quality and inaccuracy in base calling in Illumina sequencers (25). To avoid the adverse effect of the imbalance, WGBS libraries are always supplemented with a substantial amount of library of balanced nucleotide composition such as PhiX control library, or higher (G+C) content library (26), to compensate for the reduced signal intensity of C or G at the cost of reduced net WGBS throughput. To circumvent this issue and hence to fully exploit the sequencing power to WGBS, we devised a novel dual-library strategy. Therein, bisulfite-treated DNA is first divided into two fractions. One of the fractions is used to prepare a tPBAT library. Read 1 of the tPBAT library is G-poor, and hence, the library is called the ‘reverse’ library (Supplementary Table S3). The other fraction is used to prepare another tPBAT library, read 1 of which is C-poor. Hence, the library is called the ‘forward’ library (Supplementary Table S3). These two tPBAT libraries with reciprocal adaptor polarity are then combined and sequenced. Consequently, an ideal color balance should be achieved to eliminate the need for spike-in DNA, accordingly increasing the throughput.

**Figure 3.**
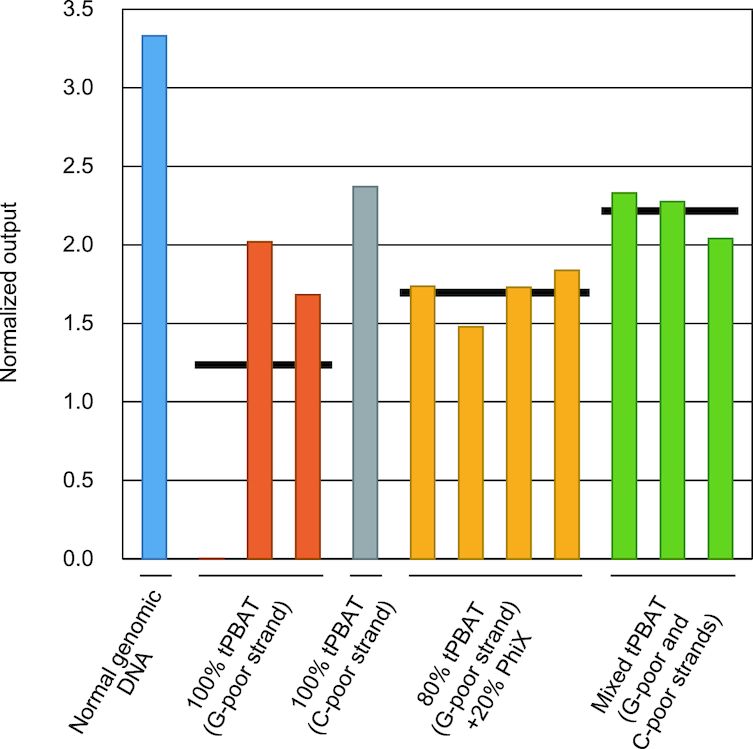
Comparison of WGBS outputs among different library mixing strategies. Two WGBS libraries with reciprocal adaptor polarity were prepared from IMR90 genomic DNA with tPBAT protocol. They were individually sequenced with or without 20% PhiX spike-in, or combinedly sequenced as indicated. We also prepared and sequenced a conventional genomic library as a control. Each dataset was obtained by a single run of Illumina MiSeq. After mapping of the reads to the human reference genome, total length of the alignments was calculated for each dataset and normalized by the cluster density (vertical axis). Black bars indicate the mean value for each mixing strategy. For details, see Supplementary Table S4.

To evaluate the power of the dual-library strategy, we used MiSeq to sequence a single tPBAT library with or without spiking-in of the PhiX control library (20%), and a mixture of two tPBAT libraries with reciprocal adaptor polarity (Supplementary Table S4). The sequencing output of single tPBAT libraries without the PhiX spike-in appeared to be unstable: one of the four sequencing runs failed completely with extremely low rates for both the pass filter and the net output of reads, and even the three successful sequencing runs were variable in terms of total alignment length per cluster density (Figure 3 and Supplementary Table S4). By contrast, single tPBAT libraries with the PhiX spike-in and the dual-library strategy showed highly consistent performance in terms of total alignment length per cluster density (Figure 3 and Supplementary Table S4). Note that the latter outperformed the former regardless of cluster density (Supplementary Table S4). We therefore concluded that the dual-library strategy was a viable choice for WGBS although the number of libraries to be prepared was doubled.

### Performance of the dual-library tPBAT on the HiSeq X Ten

Sequencing cost has been the major practical obstacle of WGBS ever since the method was introduced. However, advances in the sequencing technology led to the launch of the HiSeq X Ten system, which enabled personal genome sequencing at a cost of less than $1,000. We and others have tried to exploit the power of HiSeq X Ten for WGBS (26,27). Because of the fixed read length of the HiSeq X Ten (300 nt; 2 × 150 cycles), the WGBS library fragments would ideally exceed 300 bp. Because tPBAT has drastically improved the length of library fragment compared to rPBAT (Figure 2), it should be suitable for use with the HiSeq X Ten system. Furthermore, the dual-library has eliminated the need for control DNA spike-in (Figure 3 and Supplementary Table S4), thereby redirecting the full sequencing power of the HiSeq X Ten to WGBS.

**Figure 4.**
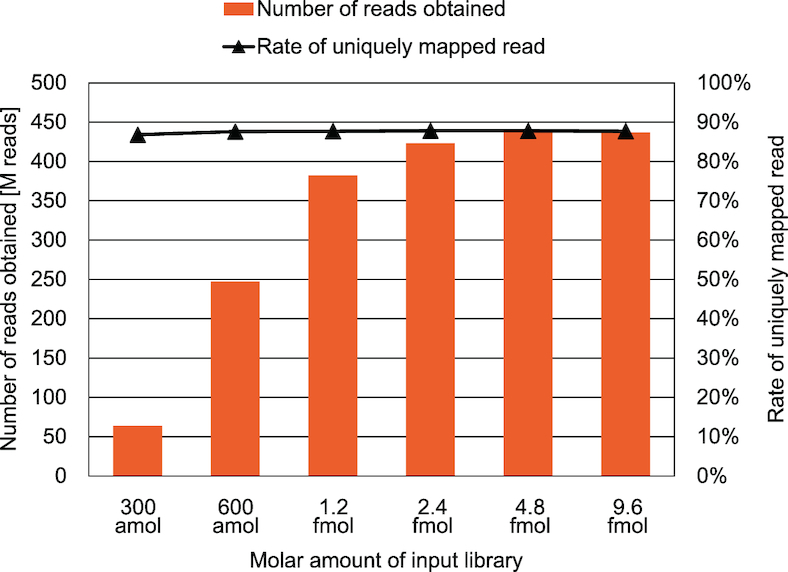
Relationship between the amount of input library and the number of reads per lane of HiSeq X Ten. Two tPBAT libraries with reciprocal adaptor polarity were combined prior to sequencing, and the indicated amount was subjected to each lane of HiSeq X Ten. We examined six different amounts of input library. The rates of uniquely mapped reads are also shown. For details, see Supplementary Table S5.

We thus conducted dual-library tPBAT of human fibroblasts IMR90 using the HiSeq X Ten system. We first used various concentrations of the tPBAT library to determine the relationship between the input DNA and the number of reads obtained with the HiSeq X Ten system because no such data were available for PBAT libraries (Figure 4 and Supplementary Table S5). As expected, the number of reads was positively correlated with the amount of injected library DNA but reached a plateau at 2.4 fmol to generate ∼430 M reads per lane (Figure 4). Importantly, the quality of the reads was comparable even when the number of reads reached the plateau (Supplementary Table S5). The deeper the genome coverage, the more accurate the estimated methylation levels. An ∼30 × coverage (i.e. ∼15 × coverage per strand) has been recommended as a standard for high-quality methylome data (28). The dual-library tPBAT achieved 27–28 × coverage per lane of the HiSeq X Ten, almost reaching the ideal WGBS coverage.

### Comparison of tPBAT with other WGBS protocols

We compared the IMR90 methylome data obtained by using tPBAT and other protocols. The tPBAT and rPBAT datasets obtained from a single lot of IMR90 genomic DNA using the same sequencing platform (HiSeq X Ten) were highly comparable in terms of methylation levels: the square of Pearson's correlation coefficients (r^2^) were 0.897 and 0.784 for 1,000-bp bin and single nucleotide resolution, respectively (Supplementary Figures S11 and 12). Comparison of these data with rPBAT data obtained using a different sequencing platform (HiSeq 2500) (11) and MethylC-seq data by Lister *et al.* (29) revealed that all these datasets were largely similar to one another in terms of methylation levels (r^2^, 0.817–0.933 for 1,000-bp bin; 0.678–0.812 for single nucleotide resolution, Supplementary Figures S11 and 12). These observations indicated that tPBAT can produce comparable methylome data with those by other protocols.

We next examined the effect of GC-content on genomic coverage. As shown in Supplementary Figure S13, MethylC-Seq (29) showed a compromised coverage of GC-rich regions, whereas rPBAT and tPBAT did a preferential coverage of GC-rich regions. A comparison between rPBAT and tPBAT data, both generated using HiSeq X Ten, revealed that the two protocols exhibited highly similar patterns of reference genome coverage (r^2^, 0.823 for 1,000-bp bin; 0.807 for single nucleotide resolution, Supplementary Figures S11 and 12), despite the difference in the principles for second round of adaptor tagging. These observations suggested that the major source of the GC-biased coverage was the initial random priming step shared by the two protocols.

We also compared two sequencing platforms, namely HiSeq X Ten and NovaSeq 6000. The latter is a current state-of-the-art sequencer expected to contribute to further reduction of sequencing cost and will be a main sequencing platform for WGBS in the near future. As shown in Supplementary Figure S14, the datasets generated by the two platforms showed concordant results in terms of both methylation levels (r^2^, 0.897 for 1,000-bp bin; 0.947 for single nucleotide resolution) and genomic coverage (r^2^, 0.891 for 1,000-bp bin; 0.892 single nucleotide resolution). We thus concluded that there is little if any difference between the two sequencing platforms.

### Performance of tPABT in low-input WGBS

**Figure 5.**
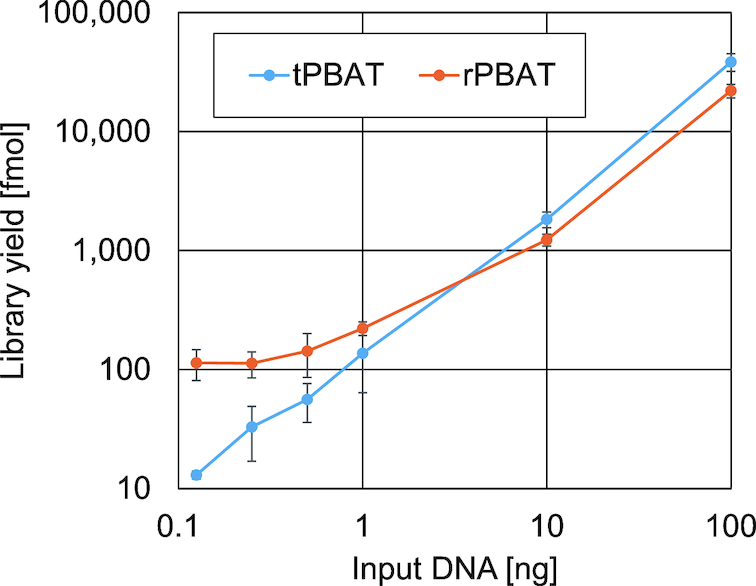
Relationship between the amount of input DNA and the molar yield of PBAT library. We prepared rPBAT and tPBAT libraries from six differential amounts of input DNA from IMR90 (*N* = 3 for each). Molar yields of individual libraries are plotted against the amounts of input DNA. For details, see Supplementary Table S7. Error bars indicate the standard deviation of three independent experiments.

Finally, we investigated the minimum amount of input DNA required for tPBAT. As shown in Figure 5, we successfully prepared tPBAT libraries from as little as 125 pg of human genomic DNA, which is equivalent to the genomic DNA from 20 human diploid cells. This indicated that the sensitivity of tPBAT was almost the same as that of rPBAT (Figure 5) (8). Molecular yields of rPBAT libraries appeared to exceed those of tPBAT libraries when starting from less than 1 ng of input DNA (Supplementary Table S7). However, the former and the latter were, respectively, larger than and equal to the expected yields linearly extrapolated from the input–yield relationship observed in the higher-input range (Figure 5). We sequenced some of these libraries to reveal that mapping rates of rPBAT and tPBAT libraries declined and remained constant, respectively, as the amount of input DNA was reduced (Supplementary Table S7). These results indicated that low-input tPBAT libraries were free from a large amount of unmappable byproducts inherent to low-input rPBAT libraries. Correction of these low-input data by their mapping rates revealed that tPBAT generated slightly fewer numbers of mappable reads than rPBAT. Nevertheless, the net outputs would be largely comparable between tPBAT and rPBAT because tPBAT outperformed rPBAT in library fragment lengths (Figure 2). Notably, tPBAT libraries maintained high mapping rates even when prepared from low-input DNA in contrast to rPBAT libraries (Supplementary Table S7). Thus, tPBAT requires a substantially smaller number of reads than rPBAT per net output: tPBAT enables more cost-effective low-input WGBS than rPBAT.

Current tPBAT requires ∼16 M reads to achieve 1 × coverage of the human genome (Supplementary Table S5), and hence, 480 M reads for 30 × coverage. Based on the relationships between the number of aligned reads and the amount of input library (Supplementary Table S5) and between the library yields and the amount of input DNA (Supplementary Table S7), we can expect that 30 ng of input DNA is sufficient for 30 × coverage of the human genome by tPBAT. Notably, the tPBAT library prepared even from 10 ng of input DNA contained more than 480 M distinct DNA molecules, which should be enough for 30 × coverage of the human genome if fully sequenced (Supplementary Table S7). It would thus be possible to obtain enough WGBS data even from a few ng of input DNA with minimum cycles of PCR amplifications to compensate for the loss of library molecules upon DNA loading on the sequencer (see conversion rate of library to reads by the HiSeq X Ten in Supplementary Table S5).

## DISCUSSION

### TACS ligation and tPBAT

In the current study, aiming to resolve the practical drawbacks of a previously devised library preparation protocol for WGBS (rPBAT), we have established a novel strategy for joining two ssDNA molecules, termed TACS ligation, and employed it for improved library preparation for WGBS (tPBAT). While highly reliable methods exist for adaptor tagging of dsDNA, adaptor tagging of ssDNA has thus far been hampered by several practical constraints. For example, TdT-mediated homopolymeric tailing is the best approach in terms of efficiency, but the homopolymer can be an obstacle for downstream sequencing. Further, while direct ligation using RNA ligases results in a ‘clean’ library structure, its efficiency is fairly limited. In addition, although random priming is efficient, it results in the shortening of the library insert. By contrast, adaptor tagging of ssDNA by TACS ligation is very efficient; TACS ligation generates an almost ‘clean’ library structure; and it does not shorten the library insert. Although the short ribonucleotide stretches created by TACS ligation necessitate the inclusion of reverse-transcriptase activity for complementary strand synthesis, Taq DNA polymerase and its variants exhibit a sufficient reverse-transcriptase activity, as demonstrated in the current study. Consequently, this potential drawback would not limit the implementation of TACS ligation. In fact, tPBAT, in which the second random priming step in conventional rPBAT protocols is replaced by TACS ligation, generated more library molecules with longer inserts than rPBAT. These results indicated that TACS ligation is indeed able to resolve one of the constraints of the original rPBAT (i.e. short insert length).

In addition to the efficiency of adaptor tagging of ssDNA, weak dependency on the 3′-terminal nucleotide base of acceptor DNA is another important feature of TACS ligation. Typically, direct joining of an adaptor to acceptor DNA using RNA ligases is more or less dependent on the 3′-terminal nucleotide of the acceptor (30). However, in TACS ligation, a few adenylates are first added to the 3′-terminus of the acceptor by a highly efficient enzyme TdT, and these common adenylate connectors, but not the 3′-terminal residue of the acceptor DNA, are used as the substrates for RNA ligase. This might explain the reduced dependency of TACS ligation on the 3′-terminal nucleobase of the acceptor. In fact, while direct joining of the adaptor to the acceptor by an RNA ligase is base-dependent, TACS ligation is unbiased in this regard (Supplementary Figure S7). Nevertheless, we observed GC content-dependent sampling bias of tPBAT was similar to rPBAT (Supplementary Figures S11–13). Consequently, the dependency on GC content might originate from the random priming reaction shared by the two protocols. To solve the GC content-dependent sampling bias of PBAT, the first random priming step in these two PBAT protocols should be replaced by other approach(es).

The extended insert length of tPBAT library resulted in enhanced sequencing output. Because the read length of the current DNA sequencers exceeds 300 nt, it is ideal to prepare libraries composed of fragments longer than 300 bp for full exploitation of the sequencing power and, hence, cost-effective sequencing. The previously developed rPBAT protocol produced a far shorter fragment library, with the peak insert size of ∼150 bp (Figure 2B and C). In the current study, the library insert was easily extended by replacing one of the two random priming steps in the original rPBAT protocol with TACS ligation (Figure 2B and C). Therefore, complete elimination of random priming from PBAT protocol is expected to elongate the library inserts to the original size of bisulfite-treated DNA (more than 500 bp) and contribute to more cost-effective methylome analysis.

### Dual-library strategy as a novel option for color compensation of WGBS

We also devised a dual-library strategy for boosting the throughput of WGBS by Illumina's sequencers (Figure 3 and Supplementary Table S4). The PhiX spike-in inevitably reduces the net WGBS throughput but avoids the risk of severe failed sequencing. By contrast, the dual-library strategy not only avoids the risk but also maintains the net WGBS throughput. Compared with the conventional 20% PhiX spike-in strategy, the dual-library strategy can enhance WGBS throughput by as much as 25%. Although the latter inevitably requires additional cost and labor for library preparation, the cost would be smaller than that of running 25% more lanes, and the labor would be substantially mitigated with automation. Given this, we believe that the dual-library strategy can be a practical option in sequencing WGBS libraries. We recognize the recently reported spike-in strategy that uses a smaller amount of control library with higher GC-content than PhiX spike-in strategy (26) as another highly practical one because every WGBS run can use the same control library prepared all at once. We nevertheless note that the beauty of a dual-library strategy over spike-in methods is its efficiency in correcting color imbalance. By simply combining the same amounts of libraries with reciprocal adaptor polarities, a dual-library strategy makes the base composition more balanced than spiking methods: the former ensures a narrower range of base/color composition than the latter (i.e. 10–40% versus 4–46%) (Supplementary Table S4).

### tPBAT as a practical low-input WGBS protocol

The current tPBAT protocol has elongated the mean library fragment length and hence made the WGBS throughput per input DNA substantially higher than the previous rPBAT protocol. Furthermore, in sharp contrast with rPBAT, tPBAT retains a high mapping rate even when applied to sub-nanogram quantities of DNA (Figure 5 and Supplementary Table S7): tPBAT enables low-input WGBS in a cost-effective manner compared to rPBAT. Therefore, it is likely that tPBAT accelerates WGBS applications to use in various samples of limited amounts, including even museum samples as well as tissues of endangered species, wherein DNA are often heavily degraded and even denatured. In contrast to MethylC-seq and Tn5mC-seq, PBAT can be applied to denatured ssDNA. Moreover, improved library fragment lengths by tPBAT should enhance WGBS of highly degraded DNA. Indeed, we have successfully applied tPBAT to library preparation from DNA extracted from formalin-fixed paraffin-embedded (FFPE) tissue sections, which is of great use in a wide variety of medical applications but unfortunately known to be heavily degraded. Although we often encountered difficulty in preparing rPBAT libraries with enough fragment lengths from FFPE samples, tPBAT has enabled us to prepare WGBS libraries even from such samples, although the fragment lengths of these libraries are inevitably shorter than those prepared from unfixed, intact genomic DNA (not shown).

Although we used in-house prepared TS2126 RNA ligase for most experiments in this study, it is not a prerequisite for tPBAT. We used it simply because development and extensive optimization of tPBAT required a large amount of this enzyme. Indeed, we have confirmed almost the same performance of tPBAT using the commercially available CircLigase II instead of the in-house prepared enzyme. We have attached a point-by-point protocol of tPBAT using CircLigase II, which we believe will help readers to reproduce tPBAT with only commercially available reagents (Supplementary Information S4).

### Future developments for further improvement of WGBS

Complete elimination of random priming from PBAT protocol is required for both reduction of the GC content-dependent sampling bias and further elongation of insert length. One may assume that direct joining of the adaptor to the 3′-terminus of bisulfite-treated DNA by TACS ligation would solve this problem. However, in our hands, application of TACS ligation to the 3′-ends of bisulfite-cleaved DNAs is currently not practical because TdT fails to modify the majority of them and because bisulfite-treated DNA cannot serve as an efficient template for Taq DNA polymerase (our unpublished observation). For the former phenomenon, we assume that DNA degradation during bisulfite treatment, presumably via cleavage in deoxyribose ring, makes the 3′-terminal structure(s) unrecognizable by TdT. For the later, we expect that DNA polymerase tolerant to uracil as well as other damaged bases would be effective. Therefore, further developments are required for a more efficient, less biased and longer-fragment library preparation for WGBS.

### TACS ligation as a widely applicable technique for efficient joining of ssDNA molecules

While we have only presented WGBS as an application of TACS ligation in the current paper, it is worth noting that TACS ligation can be used for general library preparation. Specifically, TACS ligation should be useful for library preparation from highly fragmented or degraded DNA. Because such DNA tends to be denatured or single-stranded, it cannot be tagged efficiently with adaptors using protocols designed for dsDNA. In fact, when we prepared a sequencing library from DNA extracted from micrococcal nuclease-treated nuclei, we observed improved library yield and coverage of reference genome by using a protocol that incorporated TACS ligation in comparison with a conventional kit designed for dsDNA (unpublished observation). Considering the above, we anticipate that TACS ligation will enhance the yield and quality of library preparations, especially for epigenetic analysis that involves highly degraded DNA, e.g. chromatin immunoprecipitation sequencing (ChIP-Seq), DNase l hypersensitive sites sequencing (DNase-Seq) and others.

## DATA AVAILABILITY

The sequence data used in the current study was deposited to NCBI GEO and SRA with the accession numbers listed in Supplementary Table S8. The programs are deposited in GitHub (https://github.com/FumihitoMiura/Project-2).

## Supplementary Material

gkz435_Supplemental_Files
