# Supplementary material for: Highly efficient single-stranded DNA ligation technique improves low-input whole-genome bisulfite sequencing by post-bisulfite adaptor tagging
Source: Nucleic Acids Res. 2019 May 22;47(15):e85. doi: 10.1093/nar/gkz435 (PMC6736019; doi:10.1093/nar/gkz435)
Supplement: gkz435_Supplemental_Files [file gkz435_supplemental_files.pdf]

Supplementary Data

Highly efficient single-stranded DNA ligation technique improves low-input whole-genome bisulfite sequencing by post-bisulfite adaptor tagging

Fumihito Miura[1,2 *], Yukiko Shibata[1], Miki Miura[1], Yuhei Sangatsuda[1], Osamu Hisano[1], Hiromitsu Araki[1] and Takashi Ito[1*]

[1] Department of Biochemistry, Kyushu University Graduate School of Medical Sciences, 3-1-1 Maidashi, Higashi-ku, Fukuoka 812-8582, Japan

[2] Precursory Research for Embryonic Science and Technology (PRESTO), Japan Science and Technology Agency (JST), 3-1-1 Maidashi, Higashi-ku, Fukuoka 812-8582, Japan

* To whom correspondence should be addressed. Tel: +81-92-642-6100; Fax: +81-92-642-6203; Email: fumihito@med.kyushu-u.ac.jp

Correspondence may also be addressed to tito@med.kyushu-u.ac.jp

SUPPLEMENTARY METHODS

Expression and purification of TS2126 RNA ligase

DNA fragment encoding TS2126 RNA ligase (1) was synthesized by Eurofins Genomics (Tokyo, Japan) after codon optimization for *Escherichia coli*. Recognition sequences for BamHI and EcoRI were introduced at the 5′- and 3′-ends of the protein coding sequence, respectively (Supplementary Information S1). The fragment was subcloned into the BamHI-EcoRI site of pET28a vector (Merk MilliPore, Burlington, MA). The obtained plasmid (Addgene: 76146) was then used to transform T7 Express *E. coli* (New England Biolabs, Ipswich, MA, USA). Transformants were inoculated into 3 mL of 2× YT medium (1.6% [w/v] Bacto Tryptone, 1% [w/v] Bacto yeast extract, 0.5% glucose, and 0.5% [w/v] NaCl) supplemented with 50 µg/mL of kanamycin, and grown at 37°C with shaking at 250 rpm for 16-18 h. The cultures were diluted in 1 L of 2× YT medium containing 50 µg/mL of kanamycin and incubated at 37°C with shaking at 120 rpm for 4–5 h. Protein expression was induced by the addition of isopropyl-β-D-thiogalactopyranoside (IPTG) powder (Nacalai Tesque, Kyoto, Japan) at a final concentration of 1 mM, followed by further incubation at 37°C with shaking at 120 rpm for 4 h. Cells were collected by centrifugation, suspended in 20 mL of HisTrap Buffer A (50 mM sodium phosphate, pH 7.0, 100 mM NaCl, and 10 mM imidazole), and then lysed by sonication using Digital Sonifier (Branson, Danbury, CT, USA). The cell debris was removed by centrifugation at 10,000 x *g* for 10 min, followed by filtration using 32-mm Acrodisc syringe filters with Supor Membrane (0.45-µm) (Pall, Port Washington, NY, USA). The cleared cell lysate was loaded onto AKTA start chromatography system (GE Healthcare, Pittsburgh, PA, USA) equipped with HisTrap HP column (5-mL) using an equipped template for affinity chromatography. The column was equilibrated and washed with HisTrap Buffer A, and purified protein was eluted with HisTrap Buffer B (50 mM sodium phosphate, pH 7.0, 100 mM NaCl, and 200 mM imidazole). Fractions containing the target protein were combined, diluted 20-fold with heparin buffer A (50 mM Tris-HCl, pH 8.0), and further purified using the AKTA start system equipped with HiTrap Heparin HP column (GE Healthcare). The second purification was performed using an equipped template for ion exchange chromatography with Heparin Buffer A and Heparin Buffer B (50 mM Tris-HCl, pH 8.0, and 1 M NaCl). Fractions containing the target protein were combined and then subjected to ultra-filtration on a Vivaspin 6 device with a polyethersulfone membrane of molecular weight cut-off of less than 3,000 (Sartorius, Göttingen, Germany) to exchange the buffer to storage buffer (50 mM Tris-HCl, pH 8.0, and 100 mM NaCl) and concentrate the sample. The protein concentration was determined with Protein Assay CBB Solution (Nacalai Tesque, Kyoto, Japan). After adjustment of the protein concentration to 4 mg/mL, the solution was combined with an equal volume of glycerol and stored at −20°C until use.

Expression and purification of RM378 RNA ligase

DNA fragment encoding RM378 RNA Ligase (2) was synthesized by Eurofins Genomics after codon optimization for *E. coli*. Recognition sequences for BamHI and EcoRI were introduced at the 5′- and 3′-ends of the protein coding sequence, respectively (Supplementary Information S2). The fragment was subcloned into the BamHI-EcoRI site of pColdI vector (Takara Bio Inc., Shiga, Japan). The obtained plasmid was then used to transform *E. coli* BL21 (Takara Bio Inc.). The transformants were inoculated into 3 mL of 2× YT medium containing 50 µg/mL of carbenicillin and grown at 37°C with shaking at 250 rpm for 16-18 h. Next, the cultures were diluted in 1 L of 2× YT medium containing 50 µg/mL of carbenicillin and grown with shaking at 37°C for 4–5 h. The culture was then cooled down to 16°C in ice-cold water for 30 min and protein expression was induced by the addition of IPTG powder to the final concentration at 1 mM. Then, the culture was incubated with shaking at 16°C for at least 16 h. The subsequent protein purification procedure was the same as that described for the purification of TS2126 RNA ligase.

Expression and purification of Klentaq M1 DNA polymerase

The amino acid sequence of M1 variant of full-length Taq DNA polymerase was reconstructed by referring to the description by Sauter and Marx (3), and synthesized by Eurofins Genomics after codon optimization for *E. coli.* Similarly to the two enzymes described above, recognition sequences for BamHI and EcoRI were introduced at the 5′- and 3′-ends of the protein coding sequence, respectively. The fragment was subcloned into the BamHI-EcoRI site of pColdI vector and the 5′–3′ exonuclease domain was deleted by inverse polymerase chain reaction using PrimeSTAR mutagenesis basal kit (Takara Bio Inc.). The final sequence of the insert is provided in Supplementary Information S3. The procedures for the expression and purification of the enzyme were the same as those described for the purification of RM378 RNA ligase.

Preparation of genomic DNA from IMR90 cells

The IMR90 cell line was obtained from the National Institute of Biomedical Innovation Cell Bank (Japan, JCRB9054). Cells were grown in minimum essential medium (Thermo Scientific, Waltham, MA, USA) supplemented with 10% (v/v) fetal bovine serum (GE Healthcare) and 50 U/mL Penicillin-Streptomycin (Thermo Scientific). Genomic DNA was extracted with DNeasy Blood & Tissue kit (Qiagen, Hilden, Germany) according to the manufacturer’s instructions.

Preparation of whole-genome bisulfite sequencing (WGBS) library using conventional post-bisulfite adaptor tagging (rPBAT) protocol

Library preparation using rPBAT was performed as reported previously (4). When paired-end

sequencing and/or indexing were required, another rPBAT protocol developed for targeted methylome analysis was used (5).

Library preparation from genomic DNA without bisulfite treatment

Normal genomic DNA library was prepared from 100 ng of genomic DNA extracted from IMR90 cells with ThruPLEX DNA-seq 12S Kit (Takara Bio. Inc) according to the manufacturer's instructions. The concentration was determined with a library quantitation kit from Takara Bio Inc. and sequenced with Illumina MiSeq system with version 3 kit and paired end mode (2× 75 bp) following to the manufacturer's instructions. Reads obtained were mapped on the reference genome using bowtie2 (6).

REFERENCES

1. Blondal,T., Thorisdottir,A., Unnsteinsdottir,U., Hjorleifsdottir,S., Ævarsson,A., Ernstsson,S., Fridjonsson,O.H., Skirnisdottir,S., Wheat,J.O., Hermannsdottir,A.G. *et al.* (2005) Isolation and characterization of a thermostable RNA ligase 1 from a Thermus scotoductus bacteriophage TS2126 with good single-stranded DNA ligation properties. *Nucleic Acids Res.,* **33,** 135–142.
2. Blondal,T., Hjorleifsdottir,S.H., Fridjonsson,O.F., Aevarsson,A., Skirnisdottir,S., Hermannsdottir,A.G., Hreggvidsson,G.O., Smith,A.V. and Kristjansson,J.K. (2003) Discovery and characterization of a thermostable bacteriophage RNA ligase homologous to T4 RNA ligase 1. *Nucleic Acids Res.,* **31,** 7247–7254.
3. Sauter,K.B. and Marx,A. (2006) Evolving thermostable reverse transcriptase activity in a DNA polymerase scaffold. *Angew. Chem. Int. Ed. Engl.,* **45,** 7633–7635.
4. Miura,F., Enomoto,Y., Dairiki,R. and Ito,T. (2012) Amplification-free whole-genome bisulfite sequencing by post-bisulfite adaptor tagging. Nucleic Acids Res., 40, e136.
5. Miura,F. and Ito,T. (2015) Highly sensitive targeted methylome sequencing by post-bisulfite adaptor tagging. *DNA Res.,* **22,** 13–18.
6. Langmead,B. and Salzberg,S.L. (2012) Fast gapped-read alignment with Bowtie 2. *Nature Methods,* **9,** 357.
7. Lister,R., Pelizzola,M., Dowen,R.H., Hawkins,R.D., Hon,G., Tonti-Filippini,J., Nery,J.R., Lee,L., Ye,Z., Ngo,Q.M. *et al.* (2009) Human DNA methylomes at base resolution show widespread epigenomic differences. *Nature,* **462,** 315–322.

SUPPLEMENTARY FIGURES

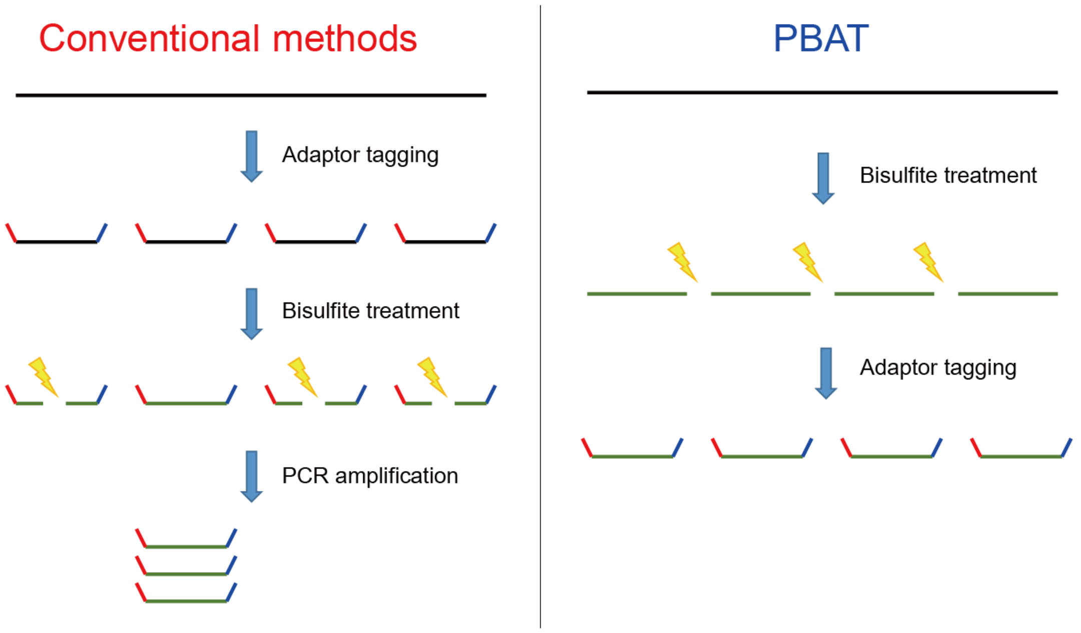

Supplementary Figure S1. PBAT enables highly efficient library preparation for whole-genome bisulfite sequencing. (Left) Conventional methods before development of PBAT first attach adaptors to both ends of DNA fragments, and then bisulfite treatment is performed. In this scheme, however, because bisulfite treatment frequently cuts DNA at random, the library molecule structure (i.e., DNA attached with adaptor sequences at both ends) will be lost. (Right) In contrast, because the adaptor tagging is performed after bisulfite treatment in the PBAT scheme, the structure of library molecules is not lost after adaptor tagging. Because bisulfite-treated DNA is single stranded, an efficient method for adaptor tagging to ssDNA is required for implementation of the PBAT scheme.

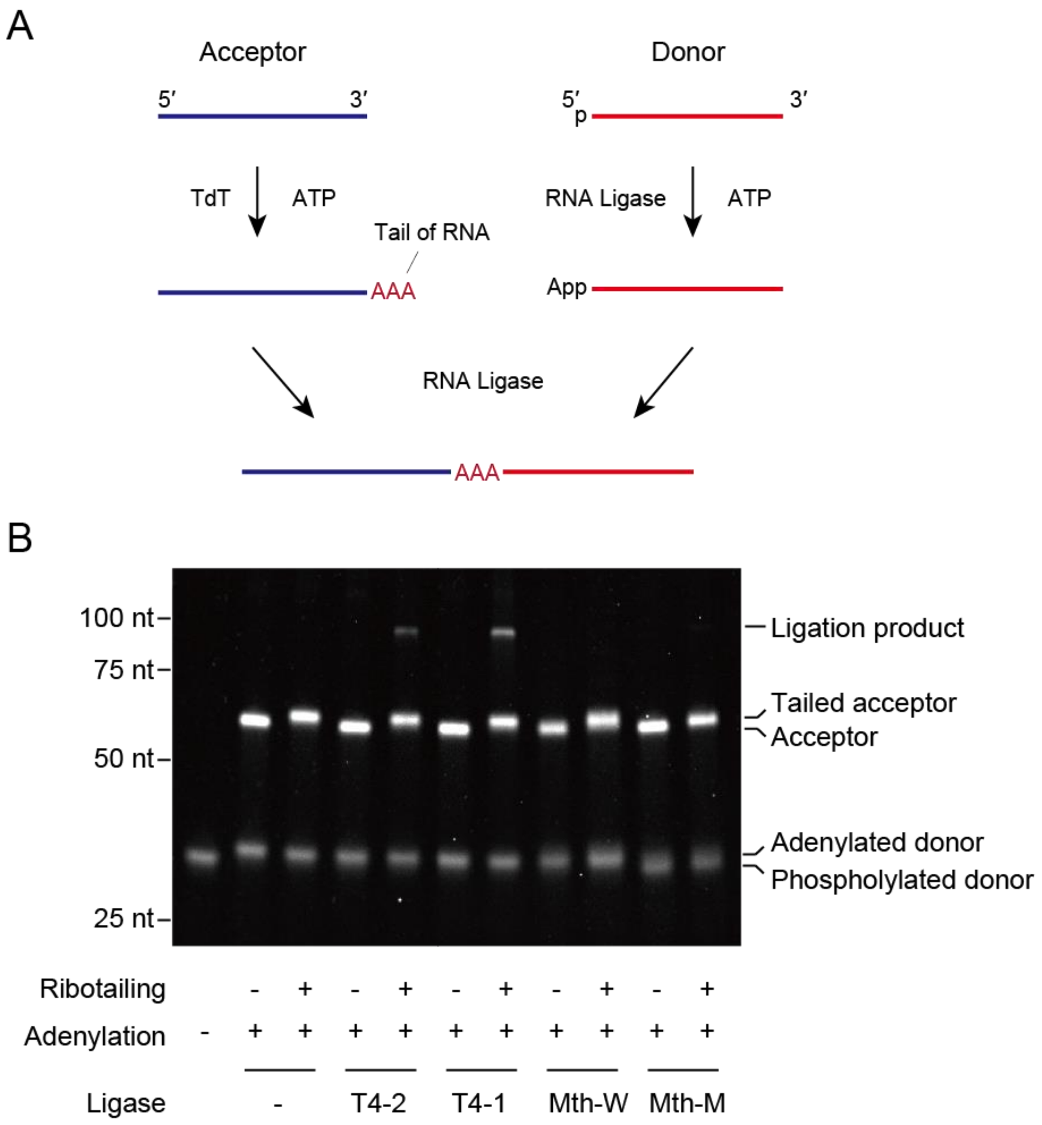

Supplementary Figure S2. TACS ligation works generally with various RNA ligases. **(**A) The experimental scheme. The competency of ligation of acceptor ODNs, with or without ribotailing by TdT and ATP, were tested with RNA ligases and a pre-adenylated donor ODN. (B) Four commercially available RNA ligases were tested in the scheme shown in (A). Donor ODN was used after adenylation by Mth RNA ligase, a component of the 5′ DNA adenylation kit (New England Biolabs). The adenylation reaction was performed in 100 µL of a solution that contained 1× TACS basal buffer [50 mM HEPES-KOH, pH 7.5, 10 mM $MgCl_2$, 0.5% Triton X-100], 1 mM ATP, 2 µM P-anti-PEA2-P (as a donor ODN, see Supplementary Table S1), and 250 pmol of Mth RNA ligase, by incubating at 60 °C for 2 h and then at 95 °C for 5 min. The ribotailing reaction was performed in 100 µL of a solution containing 1× TACS basal buffer, 1 mM ATP, 2 µM N60 (as an acceptor ODN, see Supplementary Table S1), and 200 U of TdT (Takara Bio Inc.), by

incubating at 37 °C for 2 h and then at 70 °C for 10 min. The acceptor ODN without ribotailing was prepared in the same manner, except that the reaction mixture did not contain TdT. The adenylated and ribotailed ODNs were used without further purification. The ligation reaction was performed in a 50 µL mixture that contained 1× TACS basal buffer, 100 µM ATP, 10% PEG400, 400 nM pre-adenylated donor ODN, and 400 nM acceptor ODN with or without ribotailing. For reactions with T4 RNA ligase and T4 RNA ligase 2, 40 U and 10 U of enzyme, respectively, was added to the reaction, the reaction mixtures were incubated at 25 °C for 1 h, and the enzymes were heat-inactivated at 70 °C for 10 min. For reactions with Mth RNA ligase (Mth-W) and 5′AppDNA/RNA ligase (Mth-M, New England Biolabs), 50 pmol and 20 pmol of enzyme, respectively, was added to the reaction. Next, the reaction mixtures were incubated at 65 °C for 1 h and the enzymes were heat-inactivated at 95 °C for 10 min. After the reactions, samples were analyzed using denaturing polyacrylamide gel electrophoresis with 10% Novex TBE-Urea Gel (Invitrogen). After the electrophoresis, the gel was stained with SYBR Gold Gel stain (Invitrogen) and image was obtained using a ChemiDoc system (Bio Rad Laboratories, Hercules, CA).

A

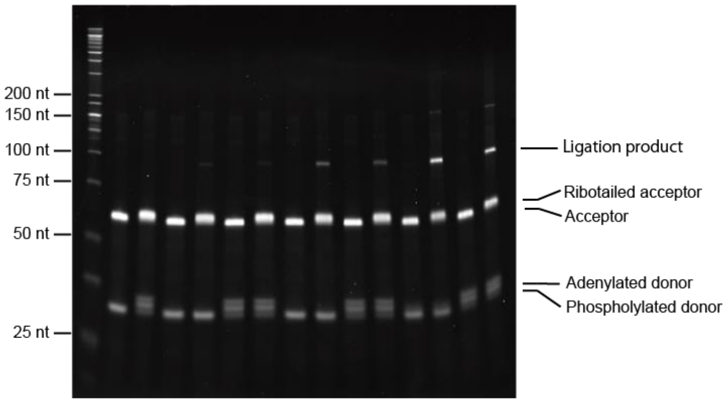

	1	2	3	4	5	6	7	8	9	10	11	12	13	14
Ribotailed acceptor		+		+		+		+		+		+		+
Acceptor	+		+		+		+		+		+		+	
Adenylated donor		+			+	+			+	+			+	+
Phospholylated donor	+		+	+			+	+			+	+		
PEG6000 (%)							5	5	5	5	10	10	10	10
T4 RNA ligase			+	+	+	+	+	+	+	+	+	+	+	+

B

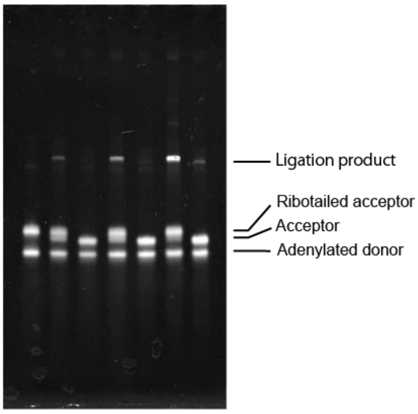

	1	2	3	4	5	6	7
Ribotailed acceptor	+	+		+		+	
Acceptor			+		+		+
Adenylated donor	+	+	+	+	+	+	+
PEG6000 (%)				5	5	10	10
5´ AppDNA/RNA ligase		+	+	+	+	+	+

Supplementary Figure S3. Ribotailing and polyethylene glycol (PEG) enhance ssDNA ligation. Ligation efficiencies were compared under varying reaction conditions. T4 RNA ligase (A) and thermostable 5′AppDNA/RNA ligase (B) were tested. Since T4 RNA ligase exhibits adenylation activity, the reaction was performed with 5′-phosphorylated and 5′-adenylated donor ODN. Conversely, because the thermostable 5′AppDNA/RNA ligase lacks the adenylation activity, only pre-adenylated donor ODN was used. Adenylation and ribotailing of donor and acceptor ODNs were achieved as described in Supplementary Figure S2, and the modified ODNs were used without further purification. The reaction was performed in 20 µL of a solution containing 1× TACS basal buffer, 250 µM ATP, 500 nM donor, 500 nM acceptor, and 40 U of T4 RNA ligase or 20 pmol

5′AppDNA/RNA ligase. The reaction mixture was incubated at 37°C for 2 h for T4 RNA ligase and 65°C for 2 h for 5′AppDNA/RNA ligase. After terminating the reaction with incubation at 95°C for 5 min, samples were analyzed using denaturing polyacrylamide gel electrophoresis as described in Supplementary Figure S2.

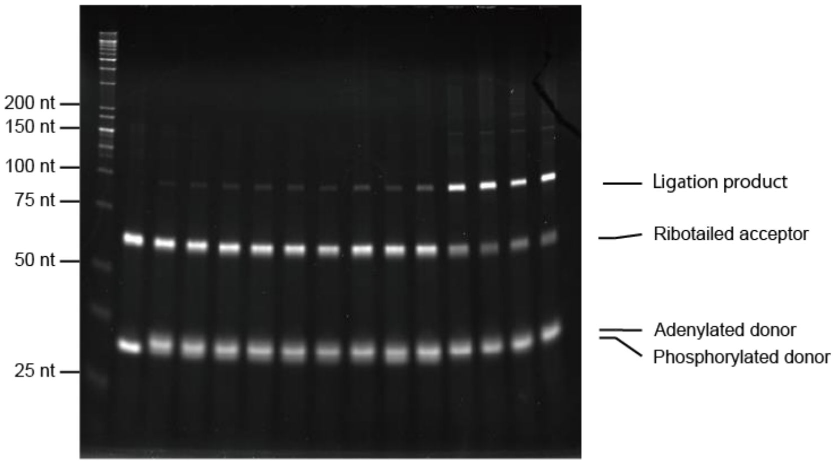

PEG6000 (%)							10						20
PEG4000 (%)						10						20	
PEG2000 (%)					10						20		
PEG1450 (%)				10						20			
PEG600 (%)			10						20				
PEG400 (%)		10						20					
TS2126 RNA Ligase	+	+	+	+	+	+	+	+	+	+	+	+	+

Supplementary Figure S4. Relationship between the efficiency of TACS ligation and molecular weight of PEG. The ability to enhance TACS ligation was compared among six PEG compounds of different molecular weights. When present at 10%, no PEG compound resulted in enhancement of TACS ligation efficiency; at 20%, the improvement was more pronounced when PEG with molecular weight higher than 1450 was used. Ribotailing of acceptor ODNs was achieved as described in Supplementary Figure S1, and the modified ODNs were used without any purification. The reaction was performed in 20 µL of a reaction solution containing 1× TACS basal buffer, 250 µM ATP, 1 µM donor (P-anti-PEA2-P), 500 nM ribotailed acceptor, and 2 µg of TS2126 RNA ligase. The reaction was performed by sequentially incubating at 65°C for 2 h and 95°C for 5 min. After the reactions, samples were analyzed using denaturing polyacrylamide gel electrophoresis as described in Supplementary Figure S2.

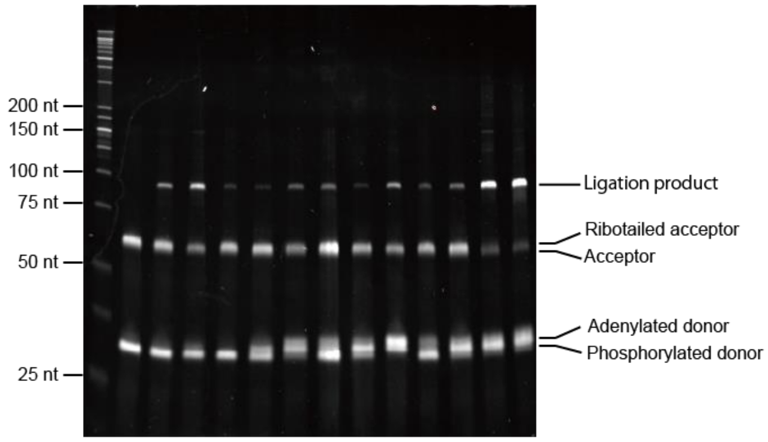

PEG6000 (%)	10	20	30	10	20	30	10	20	30	10	20	30
T4 RNA ligase	+	+	+									
RM378 RNA ligase				+	+	+						
Mth RNA ligase							+	+	+			
TS2126 RNA ligase										+	+	+

Supplementary Figure S5. TS2126 RNA ligase appears to be superior to other RNA ligases. TACS ligation activity of T4 RNA ligase (Takara Bio Inc.), RM378 RNA ligase (prepared in-house, as described in Supplementary Methods), Mth RNA ligase and TS2126 RNA ligase (prepared in-house, as described Supplementary Methods) were compared. Ribotailing of acceptor ODNs was achieved as described in Supplementary Figure S1, and the modified ODNs were used without further purification. Each reaction mixture contained 1× TACS basal buffer, 100 µM ATP, 1 µM donor (P-anti-PEA2-P), 200 nM ribotailed acceptor, and the indicated amount of PEG6000, in a total volume of 50 µL. For reactions with T4 RNA ligase, 40 U of enzyme was used, and the mixtures were incubated at 37°C for 1 h. For reactions with Mth RNA ligase, 50 pmol of enzyme was used in each reaction mixture, and the mixtures were incubated at 60°C for 1 h. For reactions with RM378 and TS2126 RNA ligase, 2 µg of the enzyme was used in each reaction mixture. The reactions were incubated at 60°C (RM378 RNA ligase) or 65°C (TS2126 RNA ligase) for 1 h. After terminating the reactions with incubation at 95°C for 5 min, samples were analyzed using denaturing polyacrylamide gel electrophoresis as described in Supplementary Figure S2.

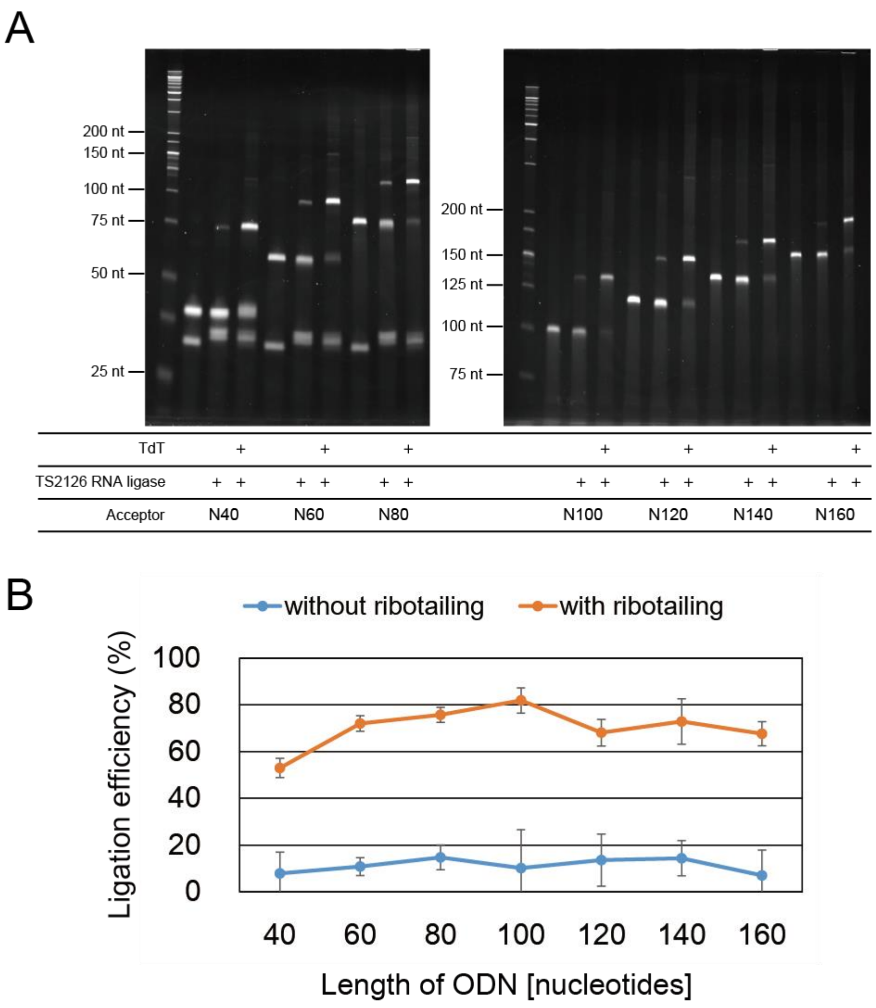

Supplementary Figure S6. TACS ligation shows minimum dependency on the size of the acceptor ODNs. Ligation efficiencies were compared between ligations with and without ribotailing using acceptor ODNs with variable lengths (N40, N60, N80, N100, N120, N140, and N160, see Supplementary Table S1). Each reaction mixture contained 1× TACS basal buffer, 50 µM ATP, 1 µM donor (P-anti-PEA2-P), 400 µM acceptor ODN, and 20% (w/v) PEG6000, in a total volume of 25 µL. The reaction mixtures contained 40 U of TdT and 2 µg of TS2126 RNA ligase when indicated. The reactions were conducted by sequentially incubating at 37°C for 30 min and 65°C for 2 h. After inactivation of enzymes with incubation at 95°C for 5 min, samples were analyzed using denaturing polyacrylamide gel electrophoresis as described in Supplementary Figure S2. (A) Representative images of gel electrophoresis. (B) Average ligation efficiencies of three independent experiments. Error bars indicate the standard deviation of three independent experiments.

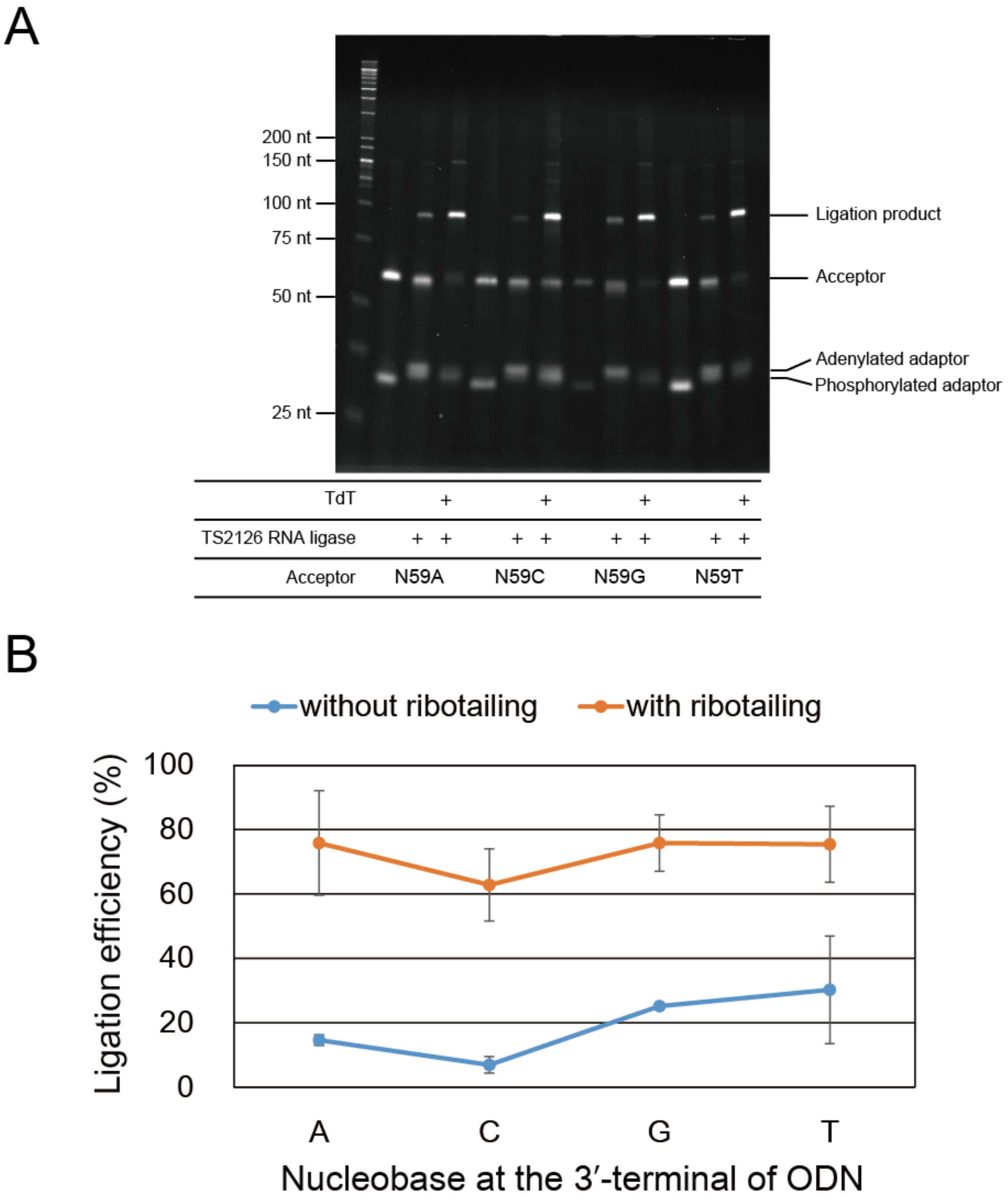

Supplementary Figure S7. TACS ligation shows less dependency on the nucleobases at the 3′-termini of the acceptor ODNs than ssDNA ligation solely dependent on RNA ligase. Ligation efficiencies were compared between ligations with and without ribotailing using ODNs of different nucleobases at their 3′ ends (N59A, N59C, N59G, and N59T, see Supplementary Table S1). Each reaction mixture contained 1× TACS basal buffer, 50 µM ATP, 1 µM donor (P-anti-PEA2-P), 400 µM acceptor ODN, and 20% (w/v) PEG6000, in a total volume of 25 µL. The reaction mixtures contained 40 U of TdT and 2 µg of TS2126 RNA ligase as indicated. The reactions were conducted by sequentially incubating at 37°C for 30 min and 65°C for 2 h. After inactivation of

enzymes with incubation at 95°C for 5 min, samples were analyzed using denaturing polyacrylamide gel electrophoresis as described in Supplementary Figure S2. (A) Representative images of gel electrophoresis. (B) Average ligation efficiencies of three independent experiments. Error bars indicate the standard deviation of three independent experiments.

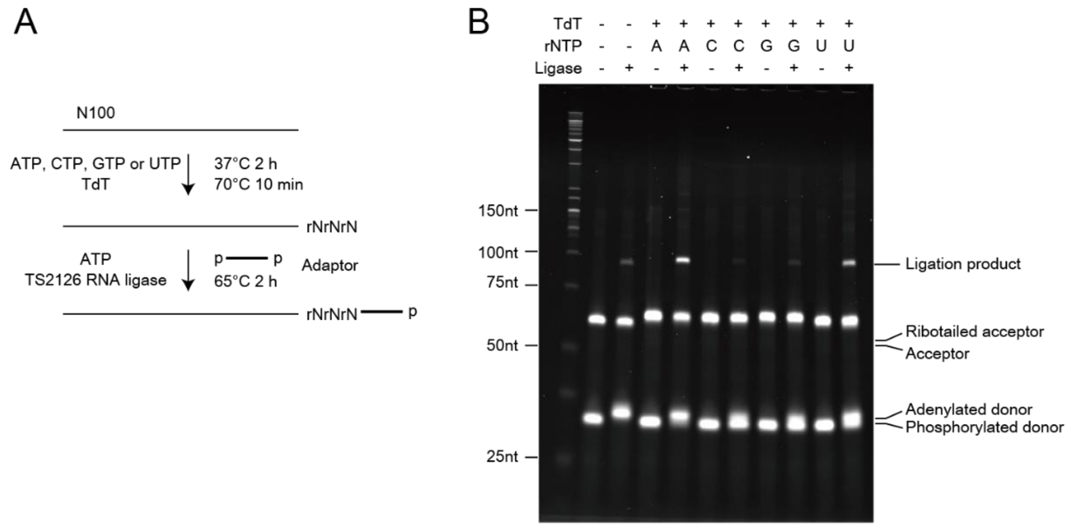

Supplementary Figure S8. Any of the four ribonucleotides can be used for ribotailing but the extent of ligation enhancement depends on the ribonucleotide used. (A) The experimental scheme. A model acceptor ODN (N100) was tailed with TdT in the presence of ATP, CTP, GTP, or UTP; ligation with a phosphorylated donor ODN was then performed. (B) Each reaction mixture contained 1×TACS basal buffer, 200 nM N100 (see Supplementary Table S1), 40 U of TdT, 1 mM of ribonucleotide triphosphate, and 20% (w/v) PEG6000, in a total volume of 50 µL. First, ribotailing was performed by incubation at 37°C for 2 h; the enzyme was then heat-inactivated by incubating at 70°C for 10 min. Next, 1 µL of 100 mM ATP and 100 pmol of donor (P-anti-PEA2-P) was added, with or without 2 µg of TS2126 RNA ligase. The reaction mixture was incubated at 65°C for 2 h. After the reactions, samples were analyzed using denaturing polyacrylamide gel electrophoresis as described in Supplementary Figure S2.

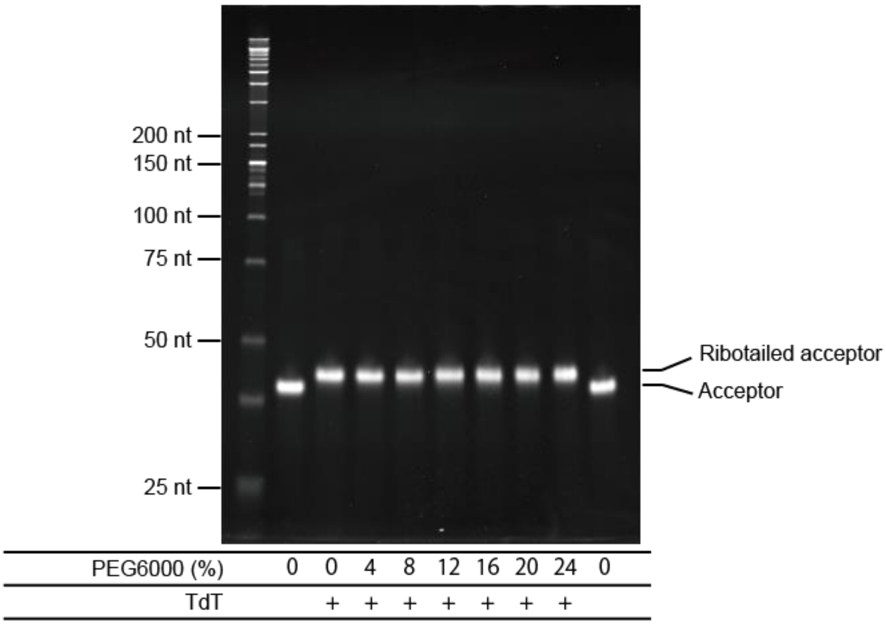

Supplementary Figure S9. The ribotailing activity of TdT is not affected by PEG. A model oligonucleotide (N40, see Supplementary Table S1) was incubated with the indicated concentration of PEG6000. The reaction mixture contained 1× TACS basal buffer, 200 nM of N40, 1 mM of ATP, 40 U of TdT (when indicated), and the indicated amount of PEG6000, in a total volume of 25 µL. The reaction mixtures were incubated at 37°C for 1 h and then at 70°C for 10 min. After the reactions, samples were analyzed using denaturing polyacrylamide gel electrophoresis as described in Supplementary Figure S2.

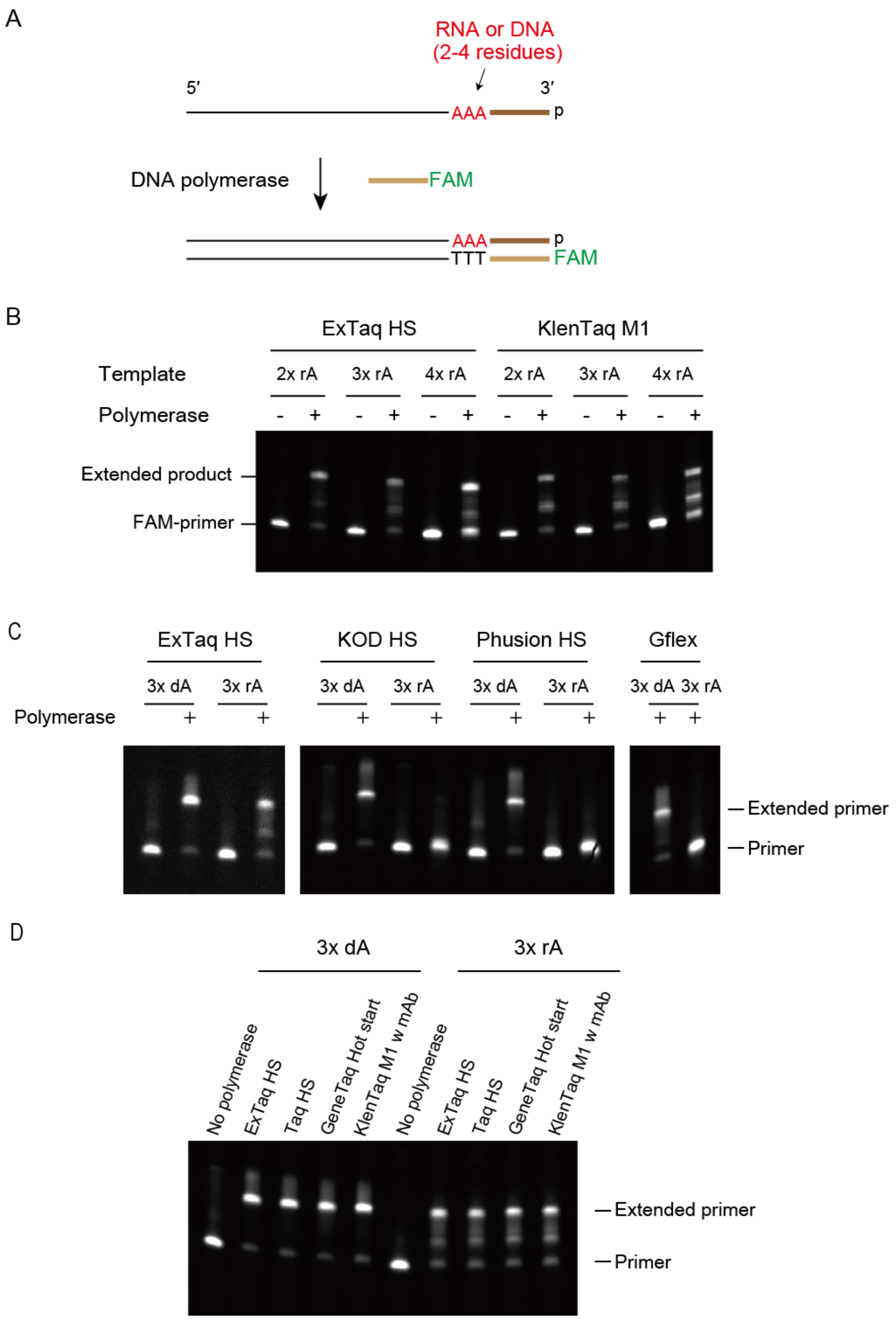

Supplementary Figure S10. Taq DNA polymerase and its variants can synthesize a sequence complementary to a short stretch of RNA at the junction generated by TACS ligation. (A) The experimental scheme. ODNs that contained 2, 3, or 4 adenylates, or 3′-deoxyadenylates

downstream of the primer-annealing site were used as model templates. A fluorescently labeled primer was annealed to the adaptor, and primer extension assays were performed. (B-D) The nucleotide sequence of model ODNs is listed in Supplementary Table S1. For templates, N10-XrA-anti-PEA2 and N10-XdA-anti-PEA2 (X denote the number of adenylates (rA) or 3′-deoxyadenylates (dA), respectively) were used, and FAM-PEA2 was used as FAM-labeled primer. Both template and primer ODNs were used at 5 µM. The number of rA or dA at the corresponding position of template ODN in (A) is indicated at the top of the gel image. The products of reaction were analyzed using denaturing polyacrylamide gel electrophoresis with 10% Nobex TBE-Urea gel. The gel images were obtained without staining; only FAM-labeled primer and its extended product(s) were detected. (B) Reverse-transcriptase activity of KlenTaq M1 polymerase (prepared in-house, as described in the Supplementary Methods) and ExTaq HS (Takara Bio Inc.) was tested. For both enzymes, a 20 µL reaction containing 1× ExTaq Buffer (Takara Bio Inc) and 250 µM dNTPs were used. Five U of ExTaq or 1 µg of KlenTaq M1 were added to the reaction and incubated at 95°C for 1 min, 55°C for 1 min, and 72°C for 3 min. (C) The ability of KOD DNA polymerase (Toyobo, Tokyo, Japan), Phusion DNA polymerase (Thermo Scientific), PrimeStar Tks Gflex DNA polymerase (Takara Bio Inc.), and ExTaq were tested to replicate an RNA-containing template. For Tks Gflex DNA polymerase, a 20 µL solution containing 1× GFlex Buffer (Takara Bio Inc.) and 1.25 unit of Tks Gflex DNA polymerase was used. For KOD plus, 20 µL of 1× KOD buffer, 250 µM of dNTPs, and 1 U of KOD Plus (Toyobo, Tokyo, Japan) was used. Each solution was incubated at 95°C for 1 min, 55°C for 1 min, and 72°C for 5 min. (D) Taq HS (Takara Bio Inc.), ExTaq HS, Hot-start Gene Taq (Nippon Gene, Toyama, Japan), and KlenTaq HS were also tested. The same reaction condition was used with ExTaq shown in (B) except for the DNA polymerase used.

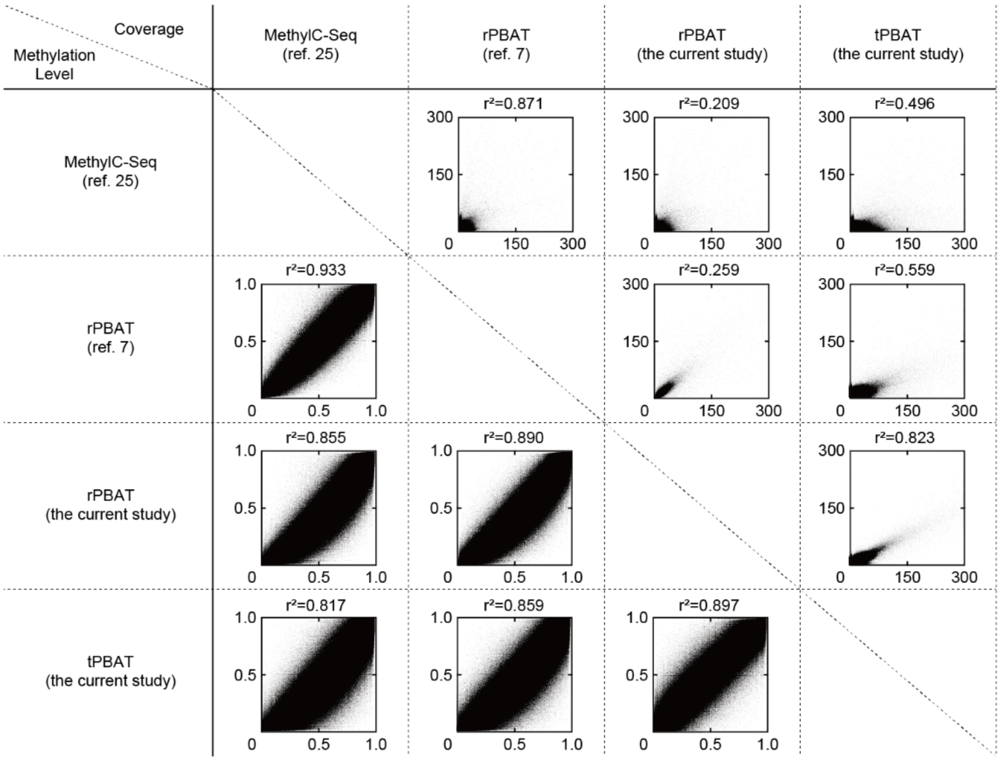

Supplementary Figure S11. Comparisons of methylome data using IMR90 cells as model. Comparison of the methylation levels and read coverage of WGBS data for 1,000-bp sliding window with 500-bp steps are shown. For each plot, the horizontal axis corresponds to the data shown in the top column, whereas the vertical axis shows data indicated in the left row. The number shown above each plot indicates the correlation coefficient between the two compared datasets. The top-right panels show the correlation of coverage; each axis indicates the coverage as a number of mapped reads. The bottom-left panels show the correlation of mean methylation levels. The basic statistics of these datasets are summarized in Supplementary Table S6. These comparisons of methylation levels and coverage between two WGBS datasets were performed using CompMethylationAnalysis (http://itolab.med.kyushu-u.ac.jp/DT/CompMethylationAnalysis/).

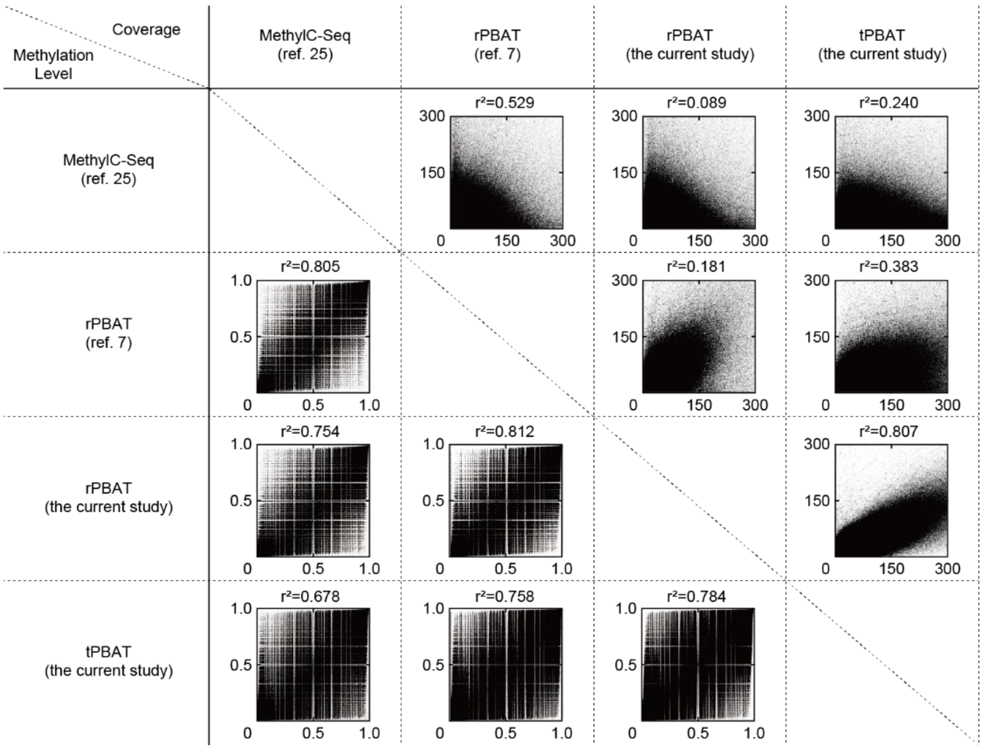

Supplementary Figure S12. Comparisons of methylome data using IMR90 cells as model. Comparison of methylation levels and read coverage of WGBS data at a single nucleotide resolution are shown as described in Supplementary Figure S11.

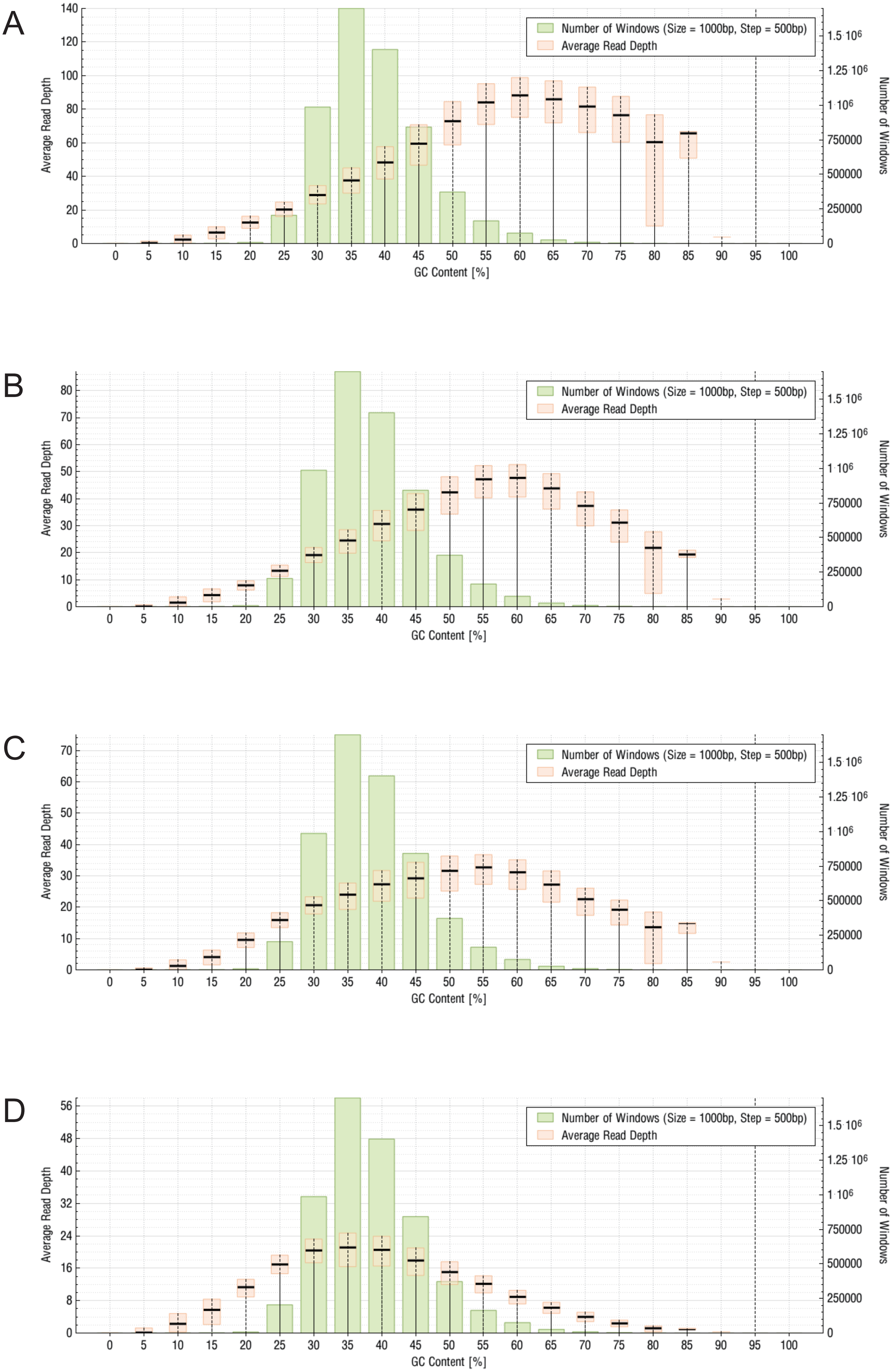
A
B
C
D
Number of Windows (Size = 1000bp, Step = 500bp)
Average Read Depth
Average Read Depth
Number of Windows
GC Content [%]

Supplementary Figure S13. Comparisons of GC content-dependent coverage of the reference genome. (A) Library prepared by tPBAT and sequenced using the HiSeq X Ten (the current study). (B) Library prepared by rPBAT from the same lot of genomic DNA as in (A) and sequenced using the HiSeq X Ten (the current study). (C) Library prepared by rPBAT and sequenced using HiSeq 2500 (5). (D) Library prepared using the MethylC-Seq protocol of Lister *et al*. (7). Horizontal axis indicates the GC content of 1,000-bp sliding window with 500-bp steps. The orange box indicates the positions of the upper and lower quartiles of the mean read coverage of each window. The interior black line of each box indicates the median of the distribution. The green bars indicate the number of 1,000-bp windows in the human reference genome.

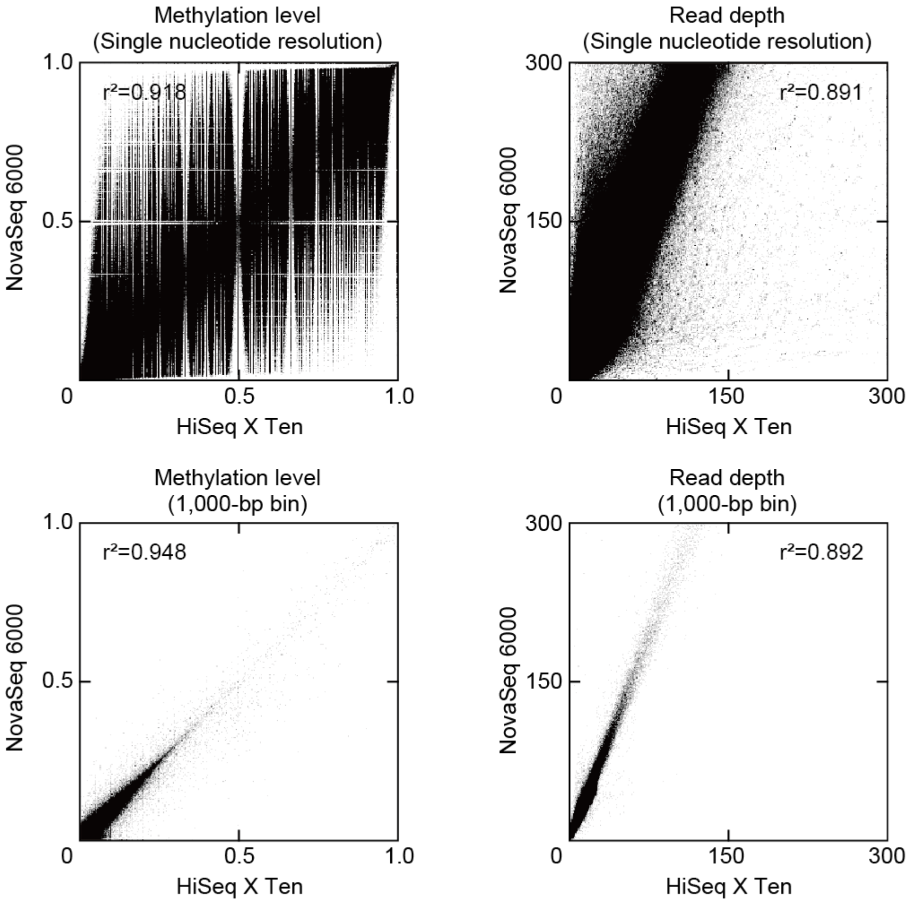

Supplementary Figure S14. Comparison of data produced by HiSeq X Ten and NovaSeq 6000. Comparisons of methylation level (left panels) and read depth (right panels) at single nucleotide resolution (top panels) and 1,000-bp bin (bottom panels) are shown. Cytosines mapped with minimum 10 reads are used for the calculations of methylation levels.

SUPPLEMENTARY TABLES

Supplementary Table S1. Oligonucleotides used in the current study

Name	Nucleotide sequence and chemical modifications
N[Number][*1]	5′-$N_{[Number]}$-3′
N59A	5′-NNNNNNNNNNNNNNNNNNNNNNNNNNNNNNNNNNNNNNNNNNNNNNNNNNNNNNNNNNNA-3′
N59C	5′-NNNNNNNNNNNNNNNNNNNNNNNNNNNNNNNNNNNNNNNNNNNNNNNNNNNNNNNNNNNC-3′
N59G	5′-NNNNNNNNNNNNNNNNNNNNNNNNNNNNNNNNNNNNNNNNNNNNNNNNNNNNNNNNNNNG-3′
N59T	5′-NNNNNNNNNNNNNNNNNNNNNNNNNNNNNNNNNNNNNNNNNNNNNNNNNNNNNNNNNNNT-3′
N10-2dA-anti-PEA2	5′-NNNNNNNNNNAAAGATCGGAAGAGCGTCGTGTAGGGAAAGAGTGT-3′
N10-3dA-anti-PEA2	5′-NNNNNNNNNNAAAAGATCGGAAGAGCGTCGTGTAGGGAAAGAGTGT-3′
N10-4dA-anti-PEA2	5′-NNNNNNNNNNAAAAAGATCGGAAGAGCGTCGTGTAGGGAAAGAGTGT-3′
N10-2rA-anti-PEA2[*2]	5′-NNNNNNNNNN[rA][rA] AGATCGGAAGAGCGTCGTGTAGGGAAAGAGTGT-3′
N10-3rA-anti-PEA2[*2]	5′-NNNNNNNNNN[rA][rA][rA]AGATCGGAAGAGCGTCGTGTAGGGAAAGAGTGT-3′
N10-4rA-anti-PEA2[*2]	5′-NNNNNNNNNN[rA][rA][rA][rA]AGATCGGAAGAGCGTCGTGTAGGGAAAGAGTGT-3′
FAM-PEA2	5′-[FAM]ACACTCTTTCCCTACACGACGCTCTTCCGATCTNNNN-3′
PEA2-N4	5′-ACACTCTTTCCCTACACGACGCTCTTCCGATCTNNNN-3′
PEA1T-N4	5′-GTGACTGGAGTTCAGACGTGTGCTCTTCCGATCTNNNN-3′
P-anti-PEA2-P	5′-[phosphate]AGATCGGAAGAGCGTCGTGTAGGGAAAGAGTGT[phosphate] -3′
PA-anti-PEA1-P	5′-[phosphate] AGATCGGAAGAGCACACGTCTGAACTCCAGTCAC[phosphate] -3′
Primer-3	5′-AATGATACGGCGACCACCGAGATCTACACACTCTTTCCCTACACGACGCTCTTCCGATCT-3′
Index-X[*2]	5′-CAAGCAGAAGACGGCATACGAGAT[Index Sequence]GTGACTGGAGTTCAGACGTGTGCTCTTCCGATC-3′

[*1] N denotes an equimolar mixture of A, C, G, and T. “Number” refers to the nucleotide length. In the current study, stretches of 40, 60, 80, 100, 120,

140, and 160 nucleotides were used.

[*2] [rA] denotes an adenosine residue.

[*3] X is an index number. A specific hexamer was inserted in the position “[Index Sequence]”. For index sequences, see Supplementary Table S2.

Supplementary Table S2. Index sequences

Index number[*1]	Index sequence[*2]	Index number[*1]	Index sequence[*2]	Index number[*1]	Index sequence[*2]
1	5′-CGTGAT-3′	9	5′-CTGATC-3′	18	5′-GCGGAC-3′
2	5′-ACATCG-3′	10	5′-AAGCTA-3′	19	5′-TTTCAC-3′
3	5′-GCCTAA-3′	11	5′-GTAGCC-3′	20	5′-GGCCAC-3′
4	5′-TGGTCA-3′	12	5′-TACAAG-3′	21	5′-CGAAAC-3′
5	5′-CACTGT-3′	13	5′-TTGACT-3′	22	5′-CGTACG-3′
6	5′-ATTGGC-3′	14	5′-GGAACT-3′	23	5′-CCACTC-3′
7	5′-GATCTG-3′	15	5′-TGACAT-3′	25	5′-ATCAGT-3′
8	5′-TCAAGT-3′	16	5′-GGACGG-3′	27	5′-AGGAAT-3′

[*1] This is the same as “X” in Supplementary Table S1.

[*2] This is the “[Index Sequence]” from Supplementary Table S1.

Supplementary Table S3. Oligonucleotide combinations for the forward and reverse libraries

Library topology	Primer for random priming	Dual-phosphorylated adaptor for TACS ligation	Synthesis of complementary strand after TACS ligation	Primer for final extension
Reverse<br>(G-poor strand in read 1)	PEA2-N4	PA-anti-PEA1-P	Index-X[*1]	Primer-3
Forward<br>(C-poor strand in read 1)	PEA1T-N4	P-anti-PEA2-P	Primer-3	Index-X[*]

[*1] X denotes the index number (see Supplementary Tables S1 and S2).

Supplementary Table S4. Comparison of sequence outputs of different library mixing strategies

Library and mixing		Normal genomic	100% tPBAT (G-poor strand)<br>(No mixing)			100% tPBAT (C-poor strand)	80% tPBAT (G-poor strand) + 20% PhiX				Mixed tPBAT (G-poor and C-poor strands)		
Calculated ideal base composition of the setup	A	30%	50%			30%	46%				40%		
	C	20%	20%			0%	20%				10%		
	G	20%	0%			20%	4%				10%		
	T	30%	30%			50%	30%				40%		
Actual base composition of read 1 (after index separation)	A	29.4%	51.7%	49.6%	49.3%	25.5%	48.4%	48.2%	48.4%	48.5%	38.6%	39.1%	39.0%
	C	20.5%	22.5%	22.9%	22.2%	2.4%	22.5%	22.3%	22.4%	22.5%	13.3%	13.7%	13.3%
	G	19.9%	22.3%	1.0%	1.0%	20.8%	2.5%	2.7%	2.2%	2.3%	10.2%	9.8%	9.8%
	T	30.3%	3.5%	26.5%	27.5%	51.2%	26.7%	26.8%	26.9%	26.7%	38.0%	37.5%	37.9%
Cluster density (K/mm^2)*1		454	473	774	936	490	544	831	588	425	385	433	872
Cluster Pass Filter		94.3%	46.1%	82.8%	74.3%	91.9%	87.7%	84.0%	85.2%	89.1%	90.5%	89.9%	85.2%
% ≥ Q30		97.3%	18.0%	93.3%	90.6%	94.1%	95.4%	93.9%	94.3%	94.7%	95.2%	94.6%	93.3%
Read pass filter (M reads)		10.4	5.3	14.9	15.36	11.01	11.2	15.9	11.8	9.1	8.42	9.33	16.9
Percent index identified		98.8%	30.1%	98.5%	99.0%	98.4%	83.1%	77.8%	86.3%	84.5%	99.1%	98.5%	98.5%
Rate for uniquely mapped reads 1*2		77.2%	1.7%	78.7%	78.1%	79.3%	74.1%	72.9%	74.4%	74.7%	78.9%	78.9%	79.0%
Rate for uniquely mapped reads 2*2		75.8%	0.6%	76.1%	73.9%	76.9%	72.9%	71.1%	72.0%	72.9%	77.6%	77.2%	76.8%
Total length of alignment (Mb)*3		1511.1	1.4	1561.9	1574.3	1160.7	943.7	1227.3	1015.9	780.1	896.9	984.6	1778.2
Total length of alignment per cluster density*4		3.328	0.003	2.018	1.682	2.369	1.735	1.477	1.728	1.836	2.330	2.274	2.039

*1 Because cluster density is difficult to adjust to the same value, the amount of input library DNA in the runs was varied.

*2 Read mapping was performed using Bowtie2 and BMap for normal human genomic library and bisulfite-treated library, respectively.

*3 Total amount of reads aligned to the reference genome.

*4 Total length of alignments divided by cluster density.

Supplementary Table S5. Relationship between input tPBAT library and sequence reads using HiSeq X Ten lane

Molar amount of input library		300 amol	600 amol	1.2 fmol	2.4 fmol	4.8 fmol	9.6 fmol
Number of template molecules served		180.6 M	361.2 M	722.4 M	1,444.8 M	2,889.6 M	5779.2 M
Number of reads obtained		63.7 M	247.2M	382.2M	423.2M	439.7M	436.7M
Conversion rate of library to reads*1		35.2%	68.4%	52.9%	29.3%	15.2%	7.5%
Rate of uniquely mapped read 1*2		86.8%	87.6%	87.7%	87.8%	87.8%	87.7%
Rate of uniquely mapped read 2*2		85.0%	85.6%	85.2%	84.9%	84.6%	84.2%
Rate of read pairs successfully mapped as a pair		67.3%	67.1%	66.8%	66.8%	66.9%	66.6%
Mean end-to-end distance of paired-end mapped reads on the reference genome		270.9 bp	282.4 bp	285.0 bp	281.3 bp	276.9 bp	273.2 bp
Mean depth (top strand)		2.0x	7.9x	12.3x	13.6x	14.1x	13.8x
Mean depth (bottom strand)		2.0x	7.9x	12.3x	13.6x	14.0x	13.8x
Mean depth (both strands)		4.0x	15.9x	24.6x	27.1x	28.1x	27.6x
Read number required to cover human genome at 1x depth		15.9 M	15.5 M	15.5 M	15.6 M	15.6 M	15.8 M
Mean methylation level (chr 1)	All C	3.6%	3.6%	3.6%	3.6%	3.6%	3.6%
	CpG	56.9%	57.6%	58.5%	59.0%	59.3%	59.4%
	CHG	1.1%	1.0%	1.0%	1.0%	1.0%	1.0%
	CHH	0.9%	0.9%	0.9%	0.9%	0.9%	0.9%
Mean methylation level (Lambda)*3	All C	0.9%	0.8%	0.8%	0.8%	0.8%	0.8%
	CpG	0.9%	0.7%	0.7%	0.7%	0.7%	0.7%
	CHG	1.1%	0.9%	0.9%	0.9%	0.9%	0.9%
	CHH	0.9%	0.8%	0.7%	0.8%	0.7%	0.7%

*1 The obtained read number was divided by the number of template molecules served.

*2 Mapping was performed using BMap in the paired-end mode.

*3 Unmethylated lambda DNA was spiked into each sample to determine the conversion rate of the bisulfite treatment.

Supplementary Table S6. Basic statistics of methylome data compared in Supplementary Figures S11–S13.

Sample Name	MethylC-Seq (ref. 7)	rPBAT (ref. 5)	rPBAT-XTen (the current study)	tPBAT-XTen (the current study)
Read of single-end sequencing or read 1 of paired-end sequencing				
Total number	1,188,017,667	953,532,455	974,278,325	673,910,633
Uniquely mapped reads	62.60%	85.70%	74.50%	86.60%
Unmapped reads	34.20%	10.30%	22.30%	9.70%
Read 2 of paired-end sequencing				
Total number	-	-	-	673,910,625
Uniquely mapped reads	-	-	-	84.60%
Unmapped reads	-	-	-	11.70%
Mean methylation rate (%)				
LAMBDA, allc	0.4	0.5	0.8	1.0
LAMBDA, CpG	0.4	0.5	0.7	0.9
LAMBDA, CHG	0.4	0.6	0.8	1.1
LAMBDA, CHH	0.4	0.5	1.0	1.0
chr1, allc	3.6	3.4	3.7	3.9
chr1, CpG	67.5	60.9	57.4	58.1
chr1, CHG	0.4	0.7	0.9	1.3
chr1, CHH	0.4	0.5	1.0	1.2
Mean read depth				
Top strand	8.8	11.5	13.2	21.5
Bottom strand	8.8	11.5	13.6	21.5
Both strands	17.6	23	26.8	43
All bases	8.8	11.5	13.4	21.5
All C	7.6	12.5	15.1	24.7
All C of CpG contexts	8.3	12.4	19.3	29.3
All C of CHG contexts	7.8	12.7	15.3	24.9
All C of CHH contexts	7.3	12.2	14.1	23.9
Median read depth				
All bases	6	10	12	18
All C	6	11	13	21
All C of CpG contexts	5	11	14	23
All C of CHG contexts	6	12	14	21
All C of CHH contexts	5	11	13	21

Supplementary Table S7. Library yields and mapping rate of reads were compared between tPBAT and rPBAT. The data presented in Figure 3 is shown with mapping rate. For yield of library, three independent preparations were summarized (mean and standard deviation are shown). For some representative conditions, sequencing was performed with Illumina MiSeq sequencer. The rates of uniquely mapped reads are shown.

Starting DNA	tPBAT[*1]			rPBAT[*1]		
	Yield (amol)	Copy number (M copies)	Mapping rate[*2]	Yield (amol)	Copy number (M copies)	Mapping rate[*2]
100 ng	38,473 ± 6,446	23,161.0	83.2%	22,014 ± 2,823	13,252.4	64.2%
10 ng	1,833 ± 281	1,103.6	-	1,230 ± 142	740.2	-
1 ng	137 ± 73	82.7	-	222 ± 29	133.5	-
500 pg	56 ± 20	33.9	-	143 ± 57	86.0	-
250 pg	33 ± 16	19.6	81.8%	113 ± 28	68.3	32.9%
125 pg	13 ± 1	7.7	76.1%	114 ± 33	68.6	17.6%

*1 Three independent experiments were performed.

*2 One of three libraries was chosen and sequenced on MiSeq with MiSeq reagent nanokit version 2. Reads were mapped as described in Materials and Methods.

Supplementary Table S8. Sequence data generated in the current study and accession numbers.

Figures and Tables used	Sequencing platforms	Sample	SRA	GEO
Figure 2 (rPBAT)	MiSeq	IMR90	SRP157972	-
Figure 2 (tPBAT)	MiSeq	IMR90	SRP157956 (SRX4553450)	-
Figure 3<br>Supplementary Table S5	MiSeq	IMR90	SRP157956	-
Figure 4<br>Supplementary Table S6	HiSeq X Ten	IMR90	SRP158894	GSE119068
Figure 5<br>Supplementary Table S7	MiSeq	IMR90	SRP186522	-
Supplementary Figures S11–S13<br>Supplementary Table S4	HiSeq X Ten	IMR90	SRP157972	GSE119069
Supplementary Figure S14	HiSeq X Ten<br>NovaSeq 6000	IMR90	SRP187153	GSE127569

SUPPLEMENTARY INFORMATION

Supplementary Information S1. Nucleotide sequence of a gene encoding codon-optimized TS2126 RNA ligase. Recognition sequences for BamHI and EcoRI are underlined.

```
   1 GGATCCATGA GCTCACTGGC TCCGTGGCGT ACGACGAGCT GGAGTCCGCT GGGCTCTCCG
  61 CCAAGTTTAG AGGATGCTTT GCGTCTTGCG CGCACAACTC GCGCATTCGC AGTCCGCCGC
 121 GATGGTGAAG GTCGCGCATT GGTTACCTAC CTGTATGGCA CTCCCGAGCT GTTCTCCCTG
 181 CCGGGCGCGC GTGAATTGCG TGGTATCGTG TATCGCGAGG AGGATGGCAC CGTGCTGAGC
 241 CGTCCGTTTC ACAAATTCTT CAACTTTGGA GAACCGTTAG CTCCGGGTGA AGAGGCCTTT
 301 AAAGCATTTC GCGATTCGAT GGTGCCCCTG TTTGTCGCCG AGAAAGTGGA TGGCTACCTG
 361 GCACAAGCGT ACCTGGATGG TGGGGAGTTG CGTTTTGCCT CTCGGCATAG CCTTAATCCG
 421 CCACTTGTGG GTGCGTTGCT GCGCAAAGCC GTCGATGAAG AAGCGATGGC GCGTCTGGGA
 481 AAACTCTTAG CTGCGGAAGG CGGGCGTTGG ACGGCCCTGT TAGAAGTGGT TGATCCGGAA
 541 GCGCCGGTCA TGGTACCGTA TCAGGAACCA GGCGTGTATC TGCTGGCCCT CCGTTCGATT
 601 GGTGAAGGGC ACTATCTTCT GCCTGGGGTA CATTTCCCGC TGCCTGAAGC CCTGCGTTAC
 661 GTTCGGTGGG AACCACGCAT GGACTTTGAC CCTCATCGCT TTCGCGGTGA AATTCGCGAC
 721 CTCCAAGGCG TAGAGGGCTA CGTGGTTACC GATGGTGCGG AGTTTGTCAA GTTCAAAACC
 781 GGCTGGGCGT TTCGGTTAGC GCGCTTCCTG ATGGACCCCG AAGGGGTGTT CCTGGAAGCC
 841 TATGCGGAAG ATCGGCTGGA CGACCTGGTG GGTGCCTTGG CGGGCCGCGA GGACCTCCTG
 901 CGTGCGGTTG CGCGTGCGCA GGATTACCTG GCAGGACTCT ATGGTGAAGC AGTTGGAGCT
 961 GGCGATGCCT TACGCCGTAT GGGCCTTCCG CGCAAGGAAG CATGGGCGCG TGTACAGGAA
1021 GAGGCCGGTC GTTGGGGCGG CTTTGCCCCT GCGTATGCTC GCGCAGCAAT GGCCGCATAT
1081 GAAGGCGGCG AAGCCCGCGA AGCGTTTCTG GTCGAACTGC GCAAACGCTC CGCTCGTAAA
1141 GCTCTGGAAG CTCTGCACTT GTTCCCACGC GTTGGTGGGG AATTACGCGG TTAAGAATTC
```

Supplementary Information S2. Nucleotide sequence of a gene encoding codon-optimized RM378 RNA ligase. Recognition sequences for BamHI and EcoRI are underlined.

```
   1 GGATCCATGG AAAGCATGAA CGTGAAGTAT CCCGTTGAGT ATTTGATAGA ACACCTGAAC
  61 TCGTTTGAAT CTCCCGAAGT TGCCGTTGAA TCACTGCGCA AAGAAGGCAT TATGTGCAAA
 121 AATCGTGGTG ATCTGTACAT GTTCAAGTAC CATCTCGGCT GCAAATTCGA CAAAATCTAC
 181 CATCTTGCAT GTCGTGGCGC GATTCTGCGC AAAACCGATT CAGGCTGGAA AGTGTTGAGC
 241 TATCCGTTTG ACAAATTCTT TAACTGGGGT GAAGAGTTGC AGCCGGAAAT TGTGAACTAC
 301 TATCAGACAC TGCGTTATGC ATCTCCTCTC AACGAGAAAC GTAAAGCGGG GTTCATGTTT
 361 AAACTGCCGA TGAAACTTGT TGAGAAGCTT GATGGTACAT GTGTCGTCTT ATACTATGAC
 421 GAGGGTTGGA AAATTCACAC CTTAGGCAGT ATTGACGCCA ATGGAAGCAT TGTCAAGAAT
 481 GGCATGGTCA CAACGCACAT GGACAAAACG TACCGCGAAC TGTTTTGGGA GACCTTTGAA
 541 AAAAAATACC CGCCTTATCT GCTGTACCAC TTGAACTCCA GCTATTGCTA CATCTTCGAA
 601 ATGGTGCATC CAGATGCCCG TGTTGTAGTA CCATATGAGG AACCCAACAT TATCCTCATT
 661 GGAGTACGCT CAGTTGATCC GGAGAAAGGG TATTTCGAAG TAGGTCCGAG TGAAGAAGCG
 721 GTTCGGATTT TCAATGAAAG CGGTGGCAAG ATCAACCTGA AACTGCCAGC TGTGCTGAGC
 781 CAAGAACAGA ACTATACTCT GTTCCGTGCA AATCGCCTAC AAGAACTGTT TGAAGAGGTC
 841 ACTCCGCTCT TCAAAAGTCT GCGTGATGGG TATGAAGTGG TGTACGAAGG CTTTGTGGCT
 901 GTTCAGGAGA TCGCTCCGAG AGTATACTAT CGCACCAAAA TCAAACATCC GGTCTATTTG
 961 GAACTGCATC GCATCAAAAC GACGATTACC CCGGAGAAAC TGGCGGACCT TTTTCTGGAG
1021 AATAAACTGG ATGATTTTGT GTTGACTCCT GATGAACAGG AAACCGTGAT GAAACTGAAG
1081 GAGATCTATA CCGATATGCG AAATCAGCTA GAATCCTCCT TCGATACGAT CTACAAAGAA
1141 ATATCGGAAC AAGTGTCGCC AGAGGAAAAT CCGGGTGAAT TTCGGAAACG CTTTGCCTTA
1201 CGCTTAATGG ACTATCATGA CAAGTCTTGG TTTTTTGCGA GGTTAGATGG TGATGAAGAA
1261 AAGATGCAGA AATCGGAGAA GAAACTGTTA ACCGAACGTA TTGAAAAAGG CCTGTTCAAA
1321 GAATTC
```

Supplementary Information S3. Nucleotide sequence of a gene encoding codon-optimized M1 variant of Taq DNA polymerase after deletion of the fragment encoding the 5′–3′ exonuclease domain (KlenTaq M1 variant). Recognition sequences for BamHI and EcoRI are underlined.

```
   1 GGATCCATGG ATGACCTTAA GCTGTCATGG GATCTTGCGA AAGTACGCAC CGATCTGCCG
  61 CTCGAAGTGG ACTTTGCTAA ACGCCGTGAA CCCGATCGCG AGCGTCTGCG CGCCTTCTTG
 121 GAACGATTGG AATTTGGTAG CCTGCTGCAT GAGTTTGGGC TTTTGGAAAG CCCGAAAGCG
 181 CTCGAAGAAG CTCCTTGGCC TCCACCCGAA GGGGCGTTTG TTGGTTTCGT ACTAAGCCGT
 241 AAAGAACCGA TGTGGGCGGA CCTGATGGCC CTGGCTGCCG CACGCGGAGG CCGCGTACAC
 301 CGTGCACCGG AACCCTACAA AGCACTGAGA GACCTCAAAG AAGCTCGCGG TCTGCTGGCC
 361 AAAGACCTGA GCGTTTTGGC ACTGCGCGAA GGACTTGGGT TACCGCCAGG AGATGATCCG
 421 ATGCTGTTAG CGTACCTCTT AGATCCGAGT AACACCACCC CGGAAGGCGT GGCGCGTCGT
 481 TATGGCGGCG AATGGACGGA GGAAGCTGGT GAACGCGCGG CCCTGTCCGA ACGCTTGTTC
 541 GCGAATCTGT GGGGCCGTCT GGAAGGCGAG GAACGACTGC TGTGGTTATA TCGTGAAGTT
 601 GAACGTCCCT TGAGTGCCGT TCTCGCGCAC ATGGAAGCGA CTGGCGTACG ACTGGACGTT
 661 GCCTACTTAC GTGCGATGTC CCTGGAGGTT GCAGAGGAAA TCGCGCGCCT TGAAGCGGAA
 721 GTTTTTCGGC TCGCCGGCCA TCCGTTCAAC CTTAATTCCC GCGATCAGCT GGAACGTGTA
 781 CTCTTCGATG AGCTTGGTTT ACCGGCTATC GGCAAAACGG AGAAAACCGG TAAACGCAGT
 841 ACGCGTGCAG CGGTGTTGGA AGCTCTGCGA GAGGCTCACC CGATTGTGGA AAAAATCCTG
 901 CAGTACCGCG AACTCACCAA ACTGAAAAGC ACCTATATTG ACCCACTGCC GGATCTGATT
 961 CACCCACGTA CGGGCCGGCT GCATACGCGG TTTAACCAGA CAGCGACGGC GACAGGGCGT
1021 CTGTCGTCTA GCGATCCGAA CCTGCAAAAT ATCCCAGTGC GCACACCGCT CGGTCAGCGT
1081 ATCAGGCGCG CTTTTATCGC CGAGGAAGGG TGGCTGCTGG TGGCACTGGA CTACTCGCAG
1141 ATTGAACTGC GTGTTCTGGC ACATCTGAGT GGCGACGAAA ACCTCATTCG CGTCTTTCAG
1201 GAGGGTCGCG ATTTTCATAC CGAAACCGCC AGCTGGATGT TCGGTGTGCC TCGGGAAGCG
1261 GTTGACCCCT TAATGCGGCG TGCCGCCAAA ACCATCAATT TCGGTGTCTT GTATGGTATG
1321 TCTGCACACC GTCTTTCACA GGAATTAGCG ATTCCATACG AAGAGGCACA AGCCTTCATC
1381 GAACGCTATT TCCAATCTTT TCCTAAAGTG CGCGCGTGGA TAGAGAAGAC CTTGGAGGAA
1441 GGACGTCGCC GCGGTTACGT TGAGACTCTG TTTGGTCGGC GGCGCTATGT GCCGGATTTA
1501 GAGGCGCGAG TCAAAGGCGT ACGTGAAGCG GCCGAACGCA TGGCATTTAA CATGCCGGTG
1561 CAAGGAACGG CAGCTGATTT GATGAAACTG GCAATGGTCA AACTGTTTCC GCGTCTTGAA
1621 GAGATGGGAG CCAGGATGTT GTTACAGGTT CACGATGAGC TCGTGTTTGA AGCGCCAAAG
1681 GAAAGAGCGG AGGCTGTCGC GCGCTTGGCG AAAGAGGTCA TGGAGGGCGT GTATCCGTTA
1741 GCGGTGCCGC TGGAAGTGGA AGTTGGCATA GGTGAGGATT GGCTGTCGGC AAAAGAGGAA
1801 TTC
```

Supplementary Information S4.

tPBAT protocol rev. 1

Reagents

- Qubit dsDNA BR Assay Kit (Thermo Fisher Scientific, Waltham, MA, # Q32850)
- Qubit ssDNA Assay Kit (Thermo Fisher Scientific, # Q10212)
- Agencourt AMPure XP (Beckman Coulter, Brea, CA, # A63880)
- Conventional 10× PCR buffer (100 mM Tris-HCl [pH 8.3], 500 mM KCl, 15 mM $MgCl_2$)
- 10 mM Tris-Acetate, pH 8.0
- 10 mM Tris-HCl, pH 8.5
- EZ DNA Methylation-Gold Kit (ZYMO Research, Irvine, CA, #D5005)
- Klenow Fragment (3′→5′ exo⁻) (New England Biolabs, Ipswich, MA, #M0212M)

  Be sure to use the enzyme provided at 10-fold higher concentration (i.e., #M0212M; 50,000 U/mL) than the conventional ones (i.e., #M0212S and #M0212L; 5,000 U/mL)

- CircLigase II ssDNA Ligase (Lucigen, Middleton, WI, # CL9021K)
- Library Quantification Kit (Takara Bio Inc., Shiga, Japan, # 638324)
- Polyethylene glycol (PEG) #400 (Nacalai Tesque, Kyoto, Japan, #11571-45)
- Polyethylene glycol (PEG) #6000 (Nacalai Tesque, #28254-85)
- 300-bp cutoff solution (50 ml)
  - PEG #400 9.5 ml (final 19%(v/v))
  - 1 M Tris-HCl, pH 8.0 0.5 ml (final 10 mM)
  - 5 M NaCl 10 ml (final 1 M)
  - ddH2O to 50 ml
- Hybridization buffer A (50 ml)
  - 5 M NaCl 9 ml (final 900 mM)
  - 1 M Tris-HCl, pH 7.4 9 ml (final 180 mM)
  - ddH2O 32 ml
- Buffer B2
  - Guanidine hydrochloride 14.3 g (final 3 M)
  - Tween 20 10 ml (final 20% (v/v))
  - ddH2O to 50 ml
- Protease K (Qiagen, Hilden, Germany, #19131)

- 2.5× TACS Buffer

➢ 1 M HEPES-KOH, pH 7.5	6.25 ml	(final 125 mM)
➢ 1 M $MgCl_2$	625 µl	(final 12.5 mM)
➢ Triton X-100	625 µl	(final 1.25%(v/v))
➢ PEG #6000	25 g	(final 50%(w/v))
➢ ddH2O	to 50 ml	

Oligonucleotides (OPC grade)

PEA2-N4	5′-ACA CTC TTT CCC TAC ACG ACG CTC TTC CGA TCT NNN N-3′
PEA1T-N4	5′-GTG ACT GGA GTT CAG ACG TGT GCT CTT CCG ATC TNN NN-3′
P-anti-PEA2-P	5′-[phosphate]AGA TCG GAA GAG CGT CGT GTA GGG AAA GAG TGT[phosphate] -3′
PA-anti-PEA1-P	5′-[phosphate] AGA TCG GAA GAG CAC ACG TCT GAA CTC CAG TCA C[phosphate] -3′
Primer-3	5′-AAT GAT ACG GCG ACC ACC GAG ATC TAC ACA CAC TCT TTC CCT ACA CGA CGC TCT TCC GAT CT-3′
Index-X (X=1-17, 18-23, 25, 27, see table below)	5′-CAA GCA GAA GAC GGC ATA CGA GAT [Index Sequence (see table below)] GTG ACT GGA GTT CAG ACG TGT GCT CTT CCG ATC-3′

Index numbers and index sequences

Index number	Index sequence	Index number	Index sequence	Index number	Index sequence
1	CGTGAT	9	CTGATC	18	GCGGAC
2	ACATCG	10	AAGCTA	19	TTTCAC
3	GCCTAA	11	GTAGCC	20	GGCCAC
4	TGGTCA	12	TACAAG	21	CGAAAC
5	CACTGT	13	TTGACT	22	CGTACG
6	ATTGGC	14	GGAACT	23	CCACTC
7	GATCTG	15	TGACAT	25	ATCAGT
8	TCAAGT	16	GGACGG	27	AGGAAT

Programs for thermal cycling

- Program 1 (for bisulfite treatment, see Section II)
  1. 98°C for 10 min
  2. 64°C for 2 h 30 min
  3. Soaking at 4°C
- Program 2 (for first-strand synthesis, see Section III)
  1. 94°C for 5 min
  2. 4°C for 5 min
  3. 4°C soak
  4. 4°C for 15 min
  5. Gradual increment from 4°C to 37°C at a rate of +1°C/min
  6. 37°C for 30 min
  7. 70°C for 10 min
  8. Soaking at 4°C
- Program 3 (for TACS ligation, see Section VII)
  1. 94°C for 3 min
  2. 4°C for 5 min
  3. 4°C soak
  4. 37°C for 30 min
  5. 65°C for 2 h
  6. 95°C for 5 min
  7. Soaking at 4°C
- Program 4 (for complementary strand synthesis, see Section VIII)
  1. 94°C for 3 min
  2. 45°C for 5 min
  3. 72°C for 30 min
  4. Soaking at 4°C

I. Estimation of DNA concentration

Accurate estimation of DNA concentration is critical. We routinely use Qubit dsDNA BR Assay Kit for the purpose. Avoid measuring OD260, since various materials other than DNA absorb 260-nm light, leading to an overestimation of DNA concentration.

1. Measure DNA concentration of sample DNA with Qubit dsDNA BR Assay Kit and Qubit Fluorometer according to the manufacturer's instruction.

II. Bisulfite treatment

We routinely start with 100 ng of DNA, because this amount is easy to handle.

1. Add 900 µl of ddH2O, 50 µl of M-dissolving buffer, and 300 µl of M-dilution buffer to one tube of CT conversion reagent.

Use freshly prepared CT conversion reagent to ensure high yield and efficient bisulfite conversion.

2. Dissolve the material by rotating the tube of CT Conversion Reagent for 10 min at room temperature.
3. Mix well the following components.

CT Conversion Reagent	130 µl
ddH2O	(20 – x) µl
Sample DNA	x µl

You may want to spike 0.5-1.0% of unmethylated lambda DNA (Promega # D1521) to this solution for monitoring bisulfite conversion rate.

4. Divide the solution into three 50-µl aliquots in PCR tubes.
5. Place the tubes on a thermal cycler, and start Program 1.
6. Place a spin column in a collection tube and add 600 µl of M-binding buffer to the column.
7. Add the sample from Step 5 to the binding buffer in the column. Close the cap and mix by inverting several times.
8. Centrifuge at full speed (≥ 10,000 × *g*) for 30 sec.
9. Reload the flow-through onto the same column again.
10. Centrifuge at full speed (≥ 10,000 × *g*) for 30 sec.

11. Discard the flow-through.
12. Add 100 µl of M-wash buffer prepared with ethanol to the column, and centrifuge at full speed for 30 sec. Discard the flow-through.
13. Add 200 µl of M-desulphonation buffer to the column and let the column stand at room temperature for 15 min.
14. Centrifuge at full speed for 30 sec. Discard the flow-through.
15. Add 200 µl of M-wash buffer with ethanol to the column and centrifuge at full speed for 30 sec. Discard the flow-through.
16. Repeat the wash in Step 15 once again, and then transfer the spin column to a new, clean 1.5-ml tube.
17. Add 22 µl of M-elution buffer directly to the column matrix and let the column stand at room temperature for 2 min. Centrifuge at full speed for 30 sec to elute the DNA.

The elution volume (22 µl) includes 1 µl for determination of yield by Qubit ssDNA Assay Kit. Typical yield of DNA is between 30% and 70% of the input. You can also analyze the bisulfite-treated DNA with denaturing gel electrophoresis by using extra 1 µl. Typical size range of the bisulfite-treated DNA is 100~1,000 nt with a peak around 600 nt.

Do not stop here. Proceed immediately to the first-strand synthesis step, as the bisulfite-treated DNA is labile.

III. First-strand synthesis

1. Prepare the first-strand synthesis reaction mix as follows in PCR tube.

10× NEBuffer 2	5 µl
2.5 mM dNTPs	5 µl
ddH2O	16 µl
PEA2-N4 (100 µM) (for reverse strand library) or<br>PEA1T-N4 (100 µM) (for forward strand library)	4 µl
Bisulfite treated sample DNA	20 µl

2. Place the tube on a thermal cycler and start Program 2. Wait until the program reaches step 3.

3. Remove the tube from the thermal cycler. Add 1 μl of Klenow fragment (exo minus) to the first-strand synthesis solution and mix well.
4. Place the tube on the thermal cycler again and proceed the Program 2 to complete the first-strand synthesis reaction.

You can stop here by either leaving the tube at 4°C or storing it at −20°C until further use. This is because the bisulfite-treated DNA is now double-stranded and excessive primers in the solution serve as a carrier DNA to prevent the adsorption of template DNA to tube wall.

IV. Removal of excess primers

1. Transfer the first strand reaction (~50 µl) into a new PCR tube, add 50 µl of AMPure XP, mix well and spin the tube briefly.

At this mixing ratio (i.e., DNA solution : AMPure XP ratio = 1:1), DNA fragments less than 200 bp are effectively removed in the supernatant. While primers and primer dimers are less than 100 nt, the products of the first-strand synthesis are larger than 200 bp.

2. Let the tube stand at room temperature for 5 min.
3. Place the tube on a magnetic stand and wait for the beads to be collected. Then, remove the supernatant carefully not to aspirate the beads.
4. Add 200 µl of 300-bp cutoff solution to the beads and vortex well.
5. Place the tube on a magnetic stand and wait for the beads to be collected. Then, remove the supernatant carefully not to aspirate the beads.
6. Repeat Step 4 and 5 once again.
7. Add 200 µl of 75% ethanol to wash the beads and then remove the supernatant.
8. Add 10 µl of 10 mM tris-acetate (pH 8.0) and vortex the tube well to disperse the beads. Following a brief centrifugation, place the tube on the magnetic stand and wait for the beads to be collected.
9. Save the supernatant in a new PCR tube.

V. TACS ligation

1. Prepare TACS ligation solution in a PCR tube as follows.

ddH2O	1.7 µl
Purified DNA from the previous step	10 µl
2.5× TACS reaction buffer	10 µl
10 mM ATP	1 µl
PA-anti-PEA1-P (100 µM) (for reverse strand library),<br>or P-anti-PEA2-P (100 µM) (for forward strand library)	0.3 µl

2. Place the tube on a thermal cycler and start the Program 3 to heat denature the double stranded DNA. Wait until the program reaches to the step 3.

Since terminal deoxynucleotidyl transferase used in following step is thermolabile, ensure that the temperature of the block reaches 4 °C.

3. Remove the PCR tube from the thermal cycler. Add following enzymes to the TACS ligation solution and mix well.

100 U/µl CircLigase II	1 µl
40 U/µl terminal deoxynucleotidyl transferase	1 µl

4. Place the tube on the thermal cycler again and proceed the Program 3 to complete the TACS ligation.

The remaining incubation steps of program 3 are 37°C for 30 min (for terminal deoxynucleotidyl transferase), 65°C for 120 min (for CircLigase II) and 95°C for 5 min (for heat-inactivation of the two enzymes).

.

VI. Complementary strand synthesis

1. Make complementary strand synthesis solution by adding following components to the TACS ligation reaction solution.

ddH2O	13.4 µl
10× PCR buffer	5 µl
2.5 mM dNTPs	4 µl
Index-X (100 µM) (for reverse strand library), or Primer-3 (100 µM) (for forward strand library)	0.6 µl
Hot Start GeneTaq	1 µl

2. Start Program 4

.

VII. DNA purification II

1. Add the following to the tube after finishing the previous step.

Buffer B2	25 μl
Protease K	5 μl

2. Incubate the tube at 50°C for 15 min.
3. Add 20 μl of AMPure XP to the solution, mix well and spin briefly.
4. Incubate for 5 min at room temperature.
5. Place the tube on a magnetic stand and wait for the beads to be separated. Then, remove the supernatant carefully not to aspirate the beads.
6. Add 200 μl of 300-bp cutoff solution to the beads and vortex well.
7. Place the tube on a magnetic stand and wait for the beads to be collected. Then, remove the supernatant carefully not to aspirate the beads.
8. Repeat step 6 and 7 once again.
9. Rinse the beads with 200 μl of 75% (v/v) ethanol.
10. Spin the tube briefly to collect residual liquid to the bottom, put the tube on magnetic stand, and remove the supernatant with a pipette.
11. Add 200 μl of 75% ethanol to wash the beads and then remove the supernatant.
12. Add 26 μl of 10 mM tris-acetate (pH 8.0) and vortex well to disperse the beads. Following a brief centrifugation, place the tube on the magnetic stand and wait for the beads to be collected.
13. Transfer the supernatant to a new PCR-tube.
14. Use 1 μl of the supernatant to measure the concentration of DNA by Qubit dsDNA HS Kit.

VIII. Final primer extension

1. Make final primer extension solution with adding followings to the DNA purified in Step IX.

ddH2O	14.7 µl
DNA purified in step VII	25 µl
10× PCR buffer	5 µl
2.5 mM dNTPs	4 µl
Primer-3 (100 µM) (for forward strand library), or Index-X (100 µM) (for reverse strand library)	0.3 µl
Hot Start GeneTaq	1 µl

2. Start Program 4

IX. DNA purification II

15. Add 50 µl of AMPure XP to the product of final primer extension reaction, mix well and spin briefly.
16. Incubate for 5 min at room temperature.
17. Place the tube on a magnetic stand and wait for the beads to be separated. Then, remove the supernatant carefully not to aspirate the beads.
18. Add 200 µl of 300-bp cutoff solution to the beads and vortex well.
19. Place the tube on a magnetic stand and wait for the beads to be collected. Then, remove the supernatant carefully not to aspirate the beads.
20. Repeat step 6 and 7 once again.
21. Rinse the beads with 200 µl of 75% (v/v) ethanol.
22. Add 22 µl of 10 mM tris-acetate (pH 8.0) and vortex well to disperse the beads. Following a brief centrifugation, place the tube on the magnetic stand and wait for the beads to be collected.
23. Transfer the supernatant to a new PCR-tube.
24. Use 1 µl of the supernatant to measure the concentration of DNA by Qubit dsDNA HS Kit.

X. qPCR quantitation of template DNA

1. Determine the exact molar concentration of template DNA using an appropriate qPCR assay.

Note that the product of Step IX contains not only intact sequencing templates but also a larger amount of byproducts. It is therefore essential to determine the correct concentration of the template DNA by qPCR, not by fluorometry. We routinely use Library Quantification Kits (CloneTech, #638324) according to the manufacturer's instructions because it is easy to use and highly reproducible. The byproducts also make it impossible to examine the size of template DNA directly by electrophoresis. Accordingly, we analyze the size of the qPCR product. Since the size distribution of PCR-amplified library on a native gel becomes unreliable after the PCR reaches to the plateau, you might consider analyzing them on a denaturing gel, such as TBE-Urea polyacrylamide gel system. Typical size is between 200 bp and 500 bp with a peak around 300 bp.
